# The compact genome of the plant pathogen *Plasmodiophora brassicae* is adapted to intracellular interactions with host *Brassica spp*

**DOI:** 10.1186/s12864-016-2597-2

**Published:** 2016-03-31

**Authors:** Stephen A. Rolfe, Stephen E. Strelkov, Matthew G. Links, Wayne E. Clarke, Stephen J. Robinson, Mohammad Djavaheri, Robert Malinowski, Parham Haddadi, Sateesh Kagale, Isobel A. P. Parkin, Ali Taheri, M. Hossein Borhan

**Affiliations:** Department of Animal and Plant Sciences, University of Sheffield, Sheffield, S10 2TN UK; Department of Agricultural, Food and Nutritional Science, University of Alberta, 410 Agriculture/Forestry Centre, Edmonton, AB T6G 2P5 Canada; Agriculture and Agri-Food Canada, 107 Science Place, Saskatoon, SK S7N 0X2 Canada; Department of Integrative Plant Biology, Institute of Plant Genetics of the Polish Academy of Sciences, ul. Strzeszynska 34, 60-479 Poznan, Poland; National Research Council Canada, 110 Gymnasium Place, Saskatoon, SK, S7N 0W9 Canada; Present address: New York Genome Center, 101 6th Ave, New York, NY 10013 USA; Present address: Department of Agricultural and Environmental Sciences, College of Agriculture, Human and Natural Sciences, Tennessee State University, 3500 John A Merritt Blvd, Nashville, TN 37209 USA

**Keywords:** *Plasmodiophora brassicae*, Clubroot, *Brassica napus*, Effectors, Biotroph

## Abstract

**Background:**

The protist *Plasmodiophora brassicae* is a soil-borne pathogen of cruciferous species and the causal agent of clubroot disease of Brassicas including agriculturally important crops such as canola/rapeseed (*Brassica napus*). *P. brassicae* has remained an enigmatic plant pathogen and is a rare example of an obligate biotroph that resides entirely inside the host plant cell. The pathogen is the cause of severe yield losses and can render infested fields unsuitable for Brassica crop growth due to the persistence of resting spores in the soil for up to 20 years.

**Results:**

To provide insight into the biology of the pathogen and its interaction with its primary host *B. napus*, we produced a draft genome of *P. brassicae* pathotypes 3 and 6 (Pb3 and Pb6) that differ in their host range. Pb3 is highly virulent on *B. napus* (but also infects other Brassica species) while Pb6 infects only vegetable Brassica crops. Both the Pb3 and Pb6 genomes are highly compact, each with a total size of 24.2 Mb, and contain less than 2 % repetitive DNA. Clustering of genome-wide single nucleotide polymorphisms (SNP) of Pb3, Pb6 and three additional re-sequenced pathotypes (Pb2, Pb5 and Pb8) shows a high degree of correlation of cluster grouping with host range. The Pb3 genome features significant reduction of intergenic space with multiple examples of overlapping untranslated regions (UTRs). Dependency on the host for essential nutrients is evident from the loss of genes for the biosynthesis of thiamine and some amino acids and the presence of a wide range of transport proteins, including some unique to *P. brassicae*. The annotated genes of Pb3 include those with a potential role in the regulation of the plant growth hormones cytokinin and auxin. The expression profile of Pb3 genes, including putative effectors, during infection and their potential role in manipulation of host defence is discussed.

**Conclusion:**

The *P. brassicae* genome sequence reveals a compact genome, a dependency of the pathogen on its host for some essential nutrients and a potential role in the regulation of host plant cytokinin and auxin. Genome annotation supported by RNA sequencing reveals significant reduction in intergenic space which, in addition to low repeat content, has likely contributed to the *P. brassicae* compact genome.

**Electronic supplementary material:**

The online version of this article (doi:10.1186/s12864-016-2597-2) contains supplementary material, which is available to authorized users.

## Background

Plasmodiophorids are obligate intracellular plant parasites that were for a long time classified as fungi but based on recent molecular phylogenies, are now classified as members of the protist subgroup Rhizaria [1]. Protists, and in particular Rhizaria, are among the least studied and poorly understood subgroups of the eukaryotes [2]. The main characteristic shared by all plasmodiophorids is cruciform nuclear division in which a persistent nucleolus elongates perpendicular to the condensed metaphase chromosomes [3]. Other common features are biflagellate zoospores, multinucleate protoplasts (plasmodia) and long-lasting resting spores [3]. Plasmodiophorids are parasites of flowering plants, brown algae, diatoms and oomycetes [4, 5]. Some plasmodiophorids cause additional damage to their host by serving as vectors for viruses. Two examples include the potato mop-top virus (PMTV) and beet necrotic yellow vein virus (BNYVV causing rhizomania of sugar beet), which are transmitted by *Spongospora subterranea* (powdery scab of potato) and *Polymyxa betae*, respectively.

One of the most economically important plasmodiophorid pathogens is *Plasmodiophora brassicae* Woronin, the causal agent of clubroot disease of cruciferous plants [6]. This disease is associated with the formation of clubs or galls on the roots of infected plants, which interfere with water and nutrient uptake leading to severe losses in crop yield and quality. Infection begins with germination of resting spores to release biflagellate primary zoospores. Motile primary zoospores swim in a film of water in the soil, attach to the root hairs and encyst. Microscopic observations by Aist and Williams [7] revealed that after formation of the cyst, the penetration of the root hair cell wall occurs through a specialized projectile-like structure (Stachel) that is formed within a tubular cavity (Rohr). About 15 min prior to penetration, the cyst vacuole enlarges, the Rohr is oriented towards the host cell wall and the end of the Rohr swells to create a structure called an Adhesorium. The Stachel then passes down the Rohr and penetrates the cell wall, which is followed by the injection of an amoeboid unit into the root hair. After entering the cell, this amoeboid unit goes through a synchronized cruciform nuclear division and forms a multinucleate primary plasmodium. Primary plasmodia cleave into secondary zoospores that are released into the soil to initiate secondary infection by penetrating the epidermis and invading the cortical tissues of the main roots. Secondary infections lead to the formation of secondary plasmodia that cause hypertrophy and hyperplasia of the infected host cells, ultimately resulting in the club-shaped malformation of the roots typical of clubroot. The secondary plasmodia cleave into resting spores, which are eventually released back into the soil when the galls decompose. The resting spores can survive in the soil for up to 20 years [8], serving as primary inoculum to initiate new infections in subsequent years.

In common with many gall-forming diseases, alterations in the concentrations of plant growth hormones, particularly cytokinin and auxin, have been proposed to play an important role in the progress of infection and development of symptoms (reviewed in [9]). Altered cytokinin and auxin concentrations are associated with the stimulation of host cell division, cell expansion and loss of differentiation in *P. brassicae-*infected tissues leading to the growth of galls. The galls are a strong metabolic sink and provide this obligate biotrophic pathogen with nutrients such as carbohydrates and amino acids. Using *Arabidopsis thaliana* as a host, Siemens and colleagues [10] reported an alteration in host cytokinin response at early stages of gall formation and a higher auxin response at later stages of infection. This was supported by monitoring the concentration of hormones and expression of phytohormone-responsive genes in roots of *A. thaliana* plants infected with *P. brassicae* [11, 12]. Recent work has shown that *P. brassicae*-induced gall formation in *A. thaliana* results from a reprogramming of host vascular cambium activity leading to increased cell proliferation, a maintenance of phloem production but a strong reduction in xylogenesis [13].

All crucifers including Brassica crops and wild species such as *A. thaliana* are potential hosts of *P. brassicae*. The disease occurs in more than 60 countries and is more prevalent in areas where Brassica crops are intensively produced such as Europe, Australia and North America [14]. Clubroot was first detected in the Canadian rapeseed or canola (*Brassica napus*) crop in 2003, with the number of *P. brassicae*-infested fields increasing significantly over the past decade [15]. Given the economic value of the canola industry to the Canadian economy (~$15 billion per annum), the spread of this disease represents a major challenge. The predominant pathotype of *P. brassicae* in Canada is pathotype 3, as classified on the differentials of Williams [16], although pathotypes 2, 5, 6 and 8 have also been identified [17–20]. Most of the research on the interaction of *P. brassicae* with its host has been limited to the plant response during infection. The soil-borne nature and biotrophic lifestyle of *P. brassicae* have hampered research on the biology of the pathogen.

To obtain insights into the biology of *P. brassicae*, we have sequenced, using a whole genome shotgun approach, the genomes of pathotypes 3 and 6 that differ in their host range and virulence, and re-sequenced pathotypes 2, 5 and 8. Comparisons were made among these novel genome sequences, and also with the recently published data from a European pathotype, e3 [21]. We combined these data with a transcriptomic approach in two host species, *B. napus* and *A. thaliana* to gain insights into the processes that underlie pathogenicity, the manipulation of host development and mechanisms of nutrient acquisition by this pathogen.

## Results and discussion

### Genome sequencing and assembly

*P. brassicae* pathotype 3 (hereafter referred to as Pb3) is the predominant strain of the clubroot pathogen in the province of Alberta in western Canada, and is highly virulent on *B. napus* but also infects other *Brassica* species [15, 17]. By contrast, pathotype 6 (Pb6) is the predominant strain of *P. brassicae* in British Columbia and Ontario, and has a host range limited to vegetable Brassicas [18]. Additional file 1: Figure S1 shows a comparison of the formation of galls upon infection of Brassica by Pb3 and Pb6.

To gain insight into the genome of *P. brassicae* and genome diversity of various pathotypes, we generated *de novo* assembled genomes of Pb3 and Pb6 and re-sequenced three additional pathotypes: Pb2, Pb5 and Pb8. Pb3 is the most virulent strain of these pathotypes [19]. Single-spore isolates of Pb3 and Pb6 [18] were propagated in the roots of their respective hosts, *B. napus* cv. DH12075 and *B. rapa* var. *pekinensis* cv. Granaat. DNA from partially purified spores was used for whole genome shotgun sequencing using a Roche 454 GS FLX Titanium platform. In addition, an 8 kb mate-paired library was sequenced for each pathotype (using a Roche GS-FLX+ platform). After removal of the host plant DNA sequences, the remaining sequence reads were assembled into 109 scaffolds for Pb3 and 356 scaffolds for Pb6 (Additional file 2: Table S1). Two Pb3 scaffolds (5 (2.522 kbp) and 38 (4.049 kbp)) were considered as potential host contamination based on 99 % identity to Brassica ribosomal RNA sequence (NCBI blastn search, E-value 0) and were excluded. The size of the assembled genome for both pathotypes was similar and approximately 24.2 Mbp, which is close to pre-existing estimates for the genome size for *P. brassicae* [22] and to the genome sequence of *P. brassicae* isolate e3 reported recently by Schwelm et al. [21]. The estimated repeat content for Pb3 and Pb6 was less than 2 %, characteristic of a small genome with few repetitive elements. The average GC content of the genome was 59 % for both Pb3 and Pb6.

The total number of predicted genes for Pb3 and Pb6 was 10,851 and 10,070, respectively. Analysis of the single-copy Core Eukaryotic Genes (CEGs) via CEGMA [23] revealed that the Pb3 genome contained 92 % complete CEGs and 93 % as partial sequences. The assembly of Pb6 yielded lower results, with 85 % of the CEGs being identified as complete and 86 % as partial.

Close examination of predicted genes and RNA sequences (from infected *B. napus* and *A. thaliana* tissue sampled at different time points after infection) mapped to the Pb3 genome revealed that the majority of the genes were located in close proximity to each other along the compact genome of *P. brassicae*. The intergenic distance for the majority of the genes was less than 800 bp (Additional file 3: Figure S2). Analysis of predicted ORFs supported by RNASeq data revealed examples of highly reduced intergenic regions with overlapping UTRs indicating shared regulatory elements for at least some of the *P. brassicae* genes (Additional file 4: Figure S3). Compact genomes and reduced intergenic space have been reported for several other eukaryotes including two unicellular algal species *Bigelowiella natans* and *Guillardia theta*, as well as the microsporidian *Antonospora locustae* [24].

The overall features reported here for the Pb3 genome are similar to the recently published genome of *P. brassicae* isolate e3 by Schwelm et al. [21]. However, the genome assembly of Pb3 was less fragmented than that of e3 (107 against 165 scaffolds) and a higher number of genes were predicted in Pb3 (10851) than in e3 (9730). A comparison of the two proteomes identified 1070 Pb3 predicted proteins that did not have an orthologue among those reported for e3 [21]. A search of Pb3 RNA sequences expressed at various stages of infection supported the presence of transcripts for 943 of these 1070 predicted genes. In addition, only five of the 1070 Pb3 genes did not have a match in the e3 genome sequence, confirming that almost all of the additional 1070 genes were present as unannotated sequences in the e3 genome assembly. Due to the importance of Pb3 as a highly prevalent and virulent pathotype infecting various Brassica crops, and also because of a more complete genome assembly (compared with Pb6 and e3), Pb3 was selected as the reference genome for further analysis.

### Genome comparison of *P. brassicae* pathotypes

**Fig. 1:**
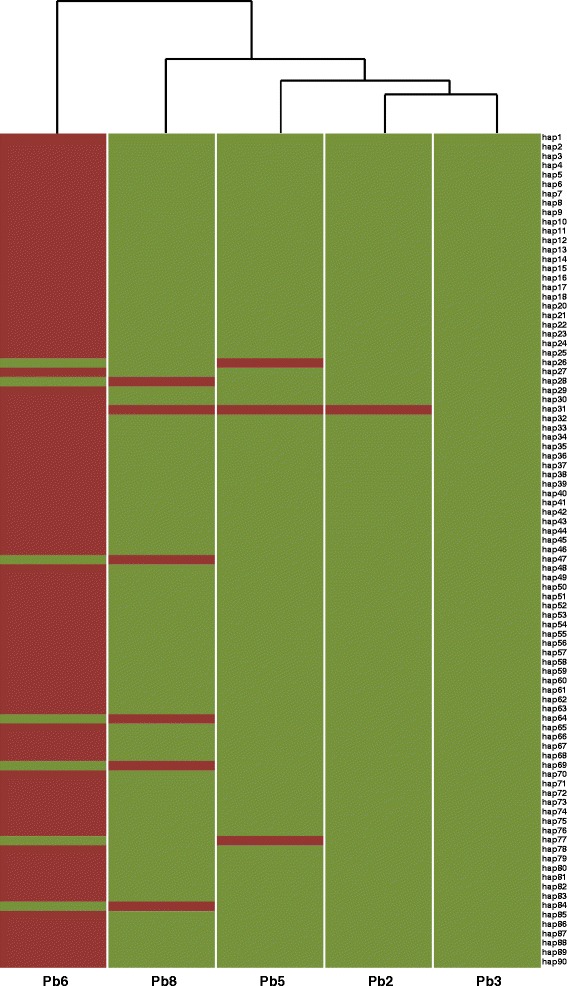
Graphical representation of the 89 SNP haplotypes discovered across four re-sequenced pathotypes. Pathotype alleles are coded in green or red for the reference (Pb3) or alternate alleles, respectively. Clustering was performed using the default dist fuction of the stats R package

Alignment of the Pb3 and Pb6 genomes revealed a close to perfect synteny with rare examples of inversion of small segments of the Pb6 genome (Additional file 5: Figure S4). The majority of predicted Pb6 genes had an orthologue in the Pb3 genome except for 14 genes that were unique to Pb6, with six of these having no homologue in the NCBI non-redundant (nr) protein database and eight that matched hypothetical proteins (E value < 0.001). To gain insight into genome variation and its correlation with host specificity we re-sequenced the genomes of Pb2, Pb5 and Pb8. A total of 173,134 SNPs were discovered by alignment of the genome sequence of Pb6 and the re-sequenced pathotypes against the Pb3 reference. Paired-end Illumina reads generated for Pb3 were also aligned to the Pb3 genome. After filtering (on allele frequency, SNP positions with heterozygous genotypes and indels) 132,429 SNPs remained and were clustered into 90 haplotype blocks. One haplotype block was found to result from a single SNP between the reference genome and Pb3 Illumina reads; investigation of this SNP indicated that it resulted from a sequencing error and this haplotype (hap19) was therefore excluded (Fig. 1). Of the remaining 89 haplotype blocks, 86.5 % contained more than one SNP. Comparison of whole genome SNP profiles revealed that Pb2, 3, 5, 8 were highly similar and clustered as one group, while the SNP profile of Pb6 was more divergent (Fig. 1 and Additional file 6: Table S2). The haplotypes correlate with the host range and geographical distribution of these pathotypes, with Pb2, 3, 5, 8 being virulent on *B. napus* and present in the prairie provinces and Pb6 failing to cause disease on *B. napus* and being prevalent in British Columbia and Eastern provinces of Canada (Additional file 7: Table S3). Without access to read data, analyses of the e3 genome could not be completed as with the other pathotypes. However, by direct sequence comparison 48,578 out of the identified 132,429 SNPs were found to differentiate the genome of e3 reported by Schwelm et al. [21] and Pb3. These SNPs were grouped into 58 haplotypes and showed that e3 clustered with Pb6 (Additional file 8: Figure S5 and Additional file 6: Table S2).

### The *P. brassicae* pathotype 3 proteome

To annotate and assign GO functions to the predicted genes, predicted Pb3 proteins were searched for homology to entries in the NCBI nr, GO and Interpro databases using Blast2Go-PRO [25]. Of the 10,851 total predicted proteins for Pb3, 3460 did not have hits in the NCBI database (*E* value < 10^−3^).

An Ortho-MCL search of protein families identified 341 paralogous groups in Pb3 with the largest groups having up to 37 paralogues. A Blast search of the NCBI nr database identified matches for only 51 of the paralogous groups. A search for orthologous proteins identified *P. ramorum* as the organism with the highest number of orthologues matching Pb3 proteins (755 Pb3 proteins). Among these were genes involved in protein and nucleic acid binding, ATP and metal ion binding, oxidoreductases, kinases and ATPase activity.

We searched the genome of Pb3 for potential secreted proteins, which might act as effectors, using the criteria described in the Methods. The total number of predicted secreted proteins was 590 (Additional file 9: Table S4), with 431 of these proteins having a probability score (D-score) of greater than 0.7. From this list, 221 proteins were defined as small (less than 300 aa) secreted proteins (SSPs). Nearly 60 % of the Pb3 SSPs (132 out of 221) had no homology with any protein in the NCBI nr database. Sixty-three of the 590 Pb3 SSPs did not have any homologue among the e3 predicted proteins (*E* value < 10^−3^). A blastn search confirmed the presence of all but one (PbPT3Sc00048_Am_3.106_1) of these genes in the e3 genome [21]. Comparing Pb3 SSPs with those predicted for Pb6 revealed that 119 of the Pb3 SSPs did not have a homologue in the Pb6 genome. Further study is required to validate the uniqueness of these 119 SPs for Pb3 and to reveal their possible role in determining host specificity. We searched the SSPs of Pb3 for the presence of RXLR motifs. RXLR motifs identified in the effectors of oomycete pathogens are believed to function in translocation of effector proteins into the plant host cells [26, 27]. RXLR type effectors are highly prevalent in *Phytophthora* and downy mildew species [26]. A potential RXLR motif was found in only 13 of the 221 predicted SSPs (Additional file 10: Table S5) indicating that this motif is not prevalent among the effectors of *P. brassicae*. The clubroot pathogen is evolutionary distinct from oomycetes and the lack or under-representation of RXLR like motifs might be expected; however, this pathogen resides within the host cell and in this regard it resembles malaria parasites (plasmodium species). Malaria parasites are also evolutionary distinct from oomycetes but have adapted a similar mechanism for translocation of effector proteins [28]. Therefore it is possible that a similar mechanism for translocation of virulent proteins operates in *P. brassicae*. RXLR motifs were also reported for some of the predicted secreted proteins from e3 [21].

### Analysis of host response through transcriptome data

Two RNASeq experiments were performed to gain insight into the genes involved in host penetration, infection and gall formation. RNA extracted from the roots of the clubroot-susceptible *B. napus* line DH12075 sampled at weekly intervals (from 1 to 6 weeks) after inoculation with Pb3 spores was sequenced. Sequences generated for RNA prepared from the roots of DH12075 plants mock inoculated with water at the same time points served as controls. In addition, we sequenced RNA from control and infected roots or hypocotyls of *A. thaliana* plants at the onset (16 DPI) and at late stages (26 DPI) of gall formation with three independent biological replicates. This second experiment used a population of *P. brassicae* described by Malinowski et al. [13].

Analysis of differential expression in infected *B. napus* found significant changes in the expression of 617 Pb3 genes during the 6-week course of infection (genes in the *B. napus* data were considered to be differentially regulated if they showed a 2-fold or greater change in expression in at least 2 consecutive samples and an adjusted *p* value of < =0.05). Within the differentially expressed genes there were genes involved in carbohydrate processing, amino-sugar and nucleotide sugar metabolism, transporters, cytoskeleton, motor proteins and flagella-associated proteins. Of the differentially regulated transcripts, 41 were genes encoding for predicted secreted proteins with 14 of them being SSPs. Hydrolases and peptidases were the most common differentially regulated secreted proteins. For the infected *A. thaliana* samples, ~25 million paired-end reads were obtained from each sample. For the 16 DPI samples, ~13 % (3.5 million) of the paired reads matched the *P. brassicae* genome in both hypocotyl and root samples. For the 26 DPI samples, 48 % of the paired reads (12 million) from hypocotyl tissue and somewhat less (34 %, 9 million) from root tissue matched the *P. brassicae* genome. The root and hypocotyl samples at each time-point were very similar to each other. Only 53 and 32 genes showed differential expression (2-fold or greater with a corrected probability of *p* ≤ 0.05) between the 16 DPI and 26 DPI hypocotyl and root tissue, respectively. In contrast, gene expression differed markedly between the 16 and 26 DPI samples within each tissue (1661 genes for hypocotyl tissue and 957 genes for root tissue, of which 763 were in common). Genes that were differentially up- or down-regulated (≥2-fold, *p* ≤ 0.05 after correction for false discovery) between 16 and 26 DPI samples in both tissue types were identified using DESeq2 and GO terms analysed using BLAST2GO. Bar charts showing GO terms that were significantly over-represented in genes that were differentially expressed between 16 and 26 DPI are shown in Additional file 11: Figure S6. Several GO terms associated with cytoskeletal and membrane activities (cytoskeletal protein binding, plasma membrane, ion transport, cytoskeleton, cell differentiation, protein transport) were down-regulated at 26 DPI compared with 16 DPI, indicative of a shift in metabolic processes associated with the change from plasmodial growth form to spore formation. Many genes associated with general cellular metabolism (carboxylic, lipid and organonitrogen metabolic processes, catalytic activity) showed the opposite pattern, with enrichment in the later stages of gall development and spore formation.

### *P. brassicae* manipulates host defence responses

A number of lines of evidence support the view that *P. brassicae,* in common with many other biotrophic pathogens, manipulates the defence responses of its hosts [9–12]. Analysis of differentially expressed putative Pb3 effectors within the first 6 weeks post-inoculation provided evidence for the manipulation of plant defence, by the induction of protease inhibitors and possibly suppression of salicylic acid (SA)-induced defence. Pairwise comparisons of *B. napus* genes differentially expressed in response to Pb3 infection using the application HORMONOMETER [29] showed that salicylic acid mediated responses at the earliest stages of infection were negatively correlated with the *A. thaliana* gene expression profile in response to SA (Additional file 12: Figure S7). In addition, the expression of a *P. brassicae* predicted secreted protein (PbPT3Sc00026_A_1.308_1) identified as a benzoic acid/salicylic acid methyltransferase (Pb3-BSMT) (NBCI accession number AFK13134) was extremely low in *B. napus* infected roots during the first week after inoculation, but increased in week 2 and peaked at weeks 3 and 4. Likewise, expression was higher in infected *A. thaliana* tissue at 16 DPI than at 26 DPI. A similar expression pattern for this gene was reported recently by Ludwig-Müller et al. [30]. The authors also confirmed the methyltransferase activity of this enzyme and suggested a possible role for this enzyme in inactivating plant defence by converting SA (Salicylic Acid) to MeSA (Methyl Salicylate), which acts as a mobile signal in systemic acquired resistance (SAR) in plants. Transient expression of Pb3-BSMT in tobacco induced SAR, as was evident by increased resistance against *Pseudomonas syringae* pv. *tabaci* in distal leaves (Additional file 13: Figure S8).

### Hallmarks of biotrophy in the *P. brassicae* genome

As on obligate biotroph, *P. brassicae* relies entirely on a living host for completion of its life cycle. A common feature of biotrophic fungal and oomycete pathogens sequenced to date is the reduction of plant cell wall degrading enzymes and inability to synthesize important metabolites [31]. We used the Kyoto Encyclopedia of Genes and Genomes (KEGG) Automated Annotation server to identify biosynthetic pathways that are present in the *P. brassicae* transcript models. We also searched manually the DNA sequence of the genome in case the transcript models were incomplete.

#### Toxin production

The genomes of obligate biotrophic plant pathogens typically have few genes associated with pathogenicity that are prevalent in necrotrophic pathogens. Many necrotrophs contain polyketide synthases (PKSs) that are involved in the production of secondary metabolites such as toxins. The genomes of the necrotrophs *Magnaporthe oryzae*, *Fusarium graminearum* and *Cochliobolus heterostrophus* are reported to encode up to 25 PKSs, but the *P. brassicae* Pb3 genome contains only one (PbPT3Sc00011_Am_2.145_1) which was expressed weakly during gall formation.

#### Cell wall degrading enzymes

**Fig. 2:**
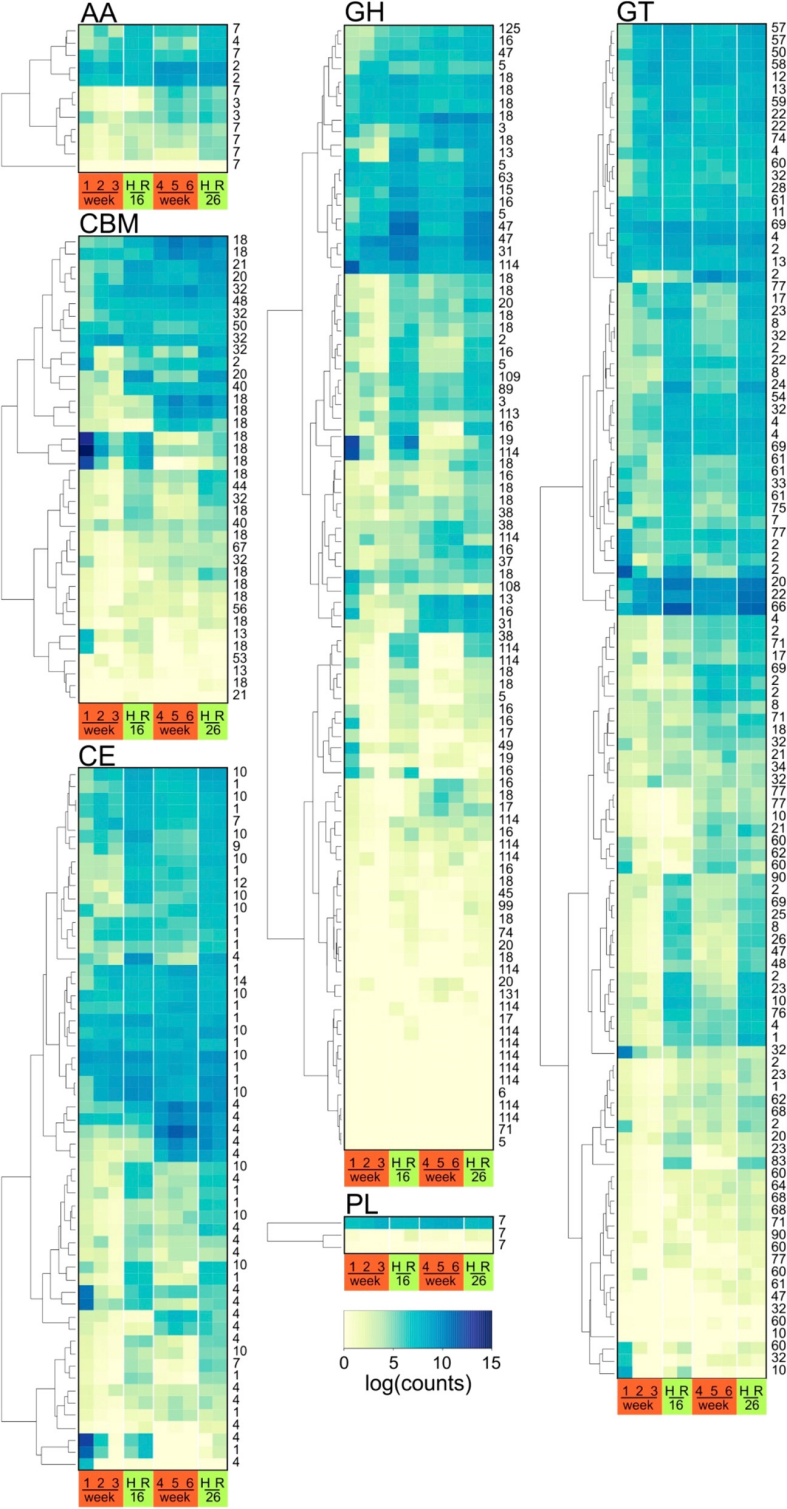
A heat map of normalised gene expression (regularised log normal expression output of DESEQ2) of carbohydrate active enzymes in infected *B. napus* (*red boxes*: weeks 1–6) and A. thaliana hypocotyl (*green boxes*) (H) and root (R) tissues at the onset (16 DPI) and late (26 DPI) stages of gall formation. Samples are ordered by similarity but sample similarity dendrograms have been omitted for clarity. AA: Auxillary activities, CBM: Carbohydrate binding modules, CE: Carbohydrate esterases, GH: Glycoside hydrolases, GT, Glycosyl transferases, PL: Pectate lyases. The numbers to the right refer to the class of carbohydrate binding modules as listed in Additional file 10: Table S5

Another feature of biotrophy is a reduction in plant cell wall degrading enzymes (CWDEs). A search of the Carbohydrate-Active EnZymes (CAZy) database for CWDEs identified 113 CWDEs enzymes in Pb3, which is nearly half of the number reported for necrotrophic and hemibiotrophic fungal plant pathogens. Figure 2 shows a heat map of normalised expression for these genes in the *B. napus* and *Arabidopsis* transcriptomes. Pb3 lacks GH61 (the copper-containing lytic polysaccharide monooxygenases), GH78 (β-glucanases) and pectate lyases (PL) of groups PL1 and PL3, which are also reported to be absent from the genome of other biotrophic pathogens [32]. The only exception was the presence of one gene (PbPT3Sc00083_Am_0.81_1) encoding for a cellobiohydrolase (GH6). The major classes of CWDEs enzymes found in the Pb3 were those for xylan (hemicellulose) degradation such as carbohydrate esterase (CE) CE1-CE4 and glycoside hydrolase (GH) family 16. Two of the most abundant GH families were GH18 and GH114 that act on chitin and chitosan, respectively. Also a carbohydrate-binding module (CBM) family 18, which binds chitin, was the most prevalent of the CBM identified in Pb3 and Pb6. All of the predicted Pb3 carbohydrate-active enzymes are listed in the Additional file 14: Table S6. Although phylogenetically distant from fungi, chitin seems to be prevalent in *P. brassicae*. Chitin is a major component (25 %) of the wall of *P. brassicae* resting spores [33]. In their analysis of the e3 clubroot genome Schwelm et al. [21] identified the importance of chitinases and chitin-binding proteins in infection.

Interestingly, one of the Pb3 genes with a predicted chitin-binding domain (PbPT3Sc00048_S_5.266_1, CBM18) was highly expressed in the first week of infection of *B. napus*, with the expression significantly reduced in week 2, and weakly expressed thereafter (weeks 3–6 after infection). A blastp search of NCBI identified a *Fusarium fujikuroi* (accession CCT72994) chitinase as the best hit (*E* value 2e-04). Fungal chitinases play a role in cell division, sporulation and spore germination among several other functions [34]. Given the timing of the expression of this Pb3 predicted chitinase, it is plausible that this gene is involved in the germination and formation of spores. In addition, there are several chitin synthases, GH family 18 and a chitin deacetylase (CDA), which were differentially expressed in *P. brassicae* during *B. napus* infection. One predicted CDA gene (PbPT3Sc00039_A_4.359_1, CE4) was significantly up-regulated in weeks 3–6 after inoculation (coinciding with the production and release of resting spores). Another gene (PbPT3Sc00039_Sm_3.268_1, CE4) predicted as a CDA/polysaccharide deacetylase was significantly up-regulated in the first week of infection. In fungi, it has been suggested that modification of surface chitin by CDA could result in protection of the cell wall from enzymatic hydrolysis, and help to avoid generation of an auto-catalytic defence response system in the invaded host plant [35]. Collectively, these data provide a picture of the fine regulation of genes involved in the synthesis, turn-over (hydrolysis) and modification (deacetylation to protect chitin containing cell walls from degradation) of chitin in *P. brassicae*, which correlates with the various life stages of the pathogen.

#### Nutrient acquisition

The *P. brassicae* genome contains a complete molybdopterin biosynthetic pathway but the thiamine biosynthetic pathway is absent. Eleven putative thiamine transporters were identified within the Pb3 genome, consistent with the acquisition of this essential vitamin from its host. The genome encoded a sulfite oxidase but nitrate reductase was missing, with nitrogen acquired from the host in more complex forms.

Additional file 15: Table S7 shows the biosynthetic pathways for amino acids and their relative completeness in the *P. brassicae* genome. The shikimic acid pathway to chorismate was complete with incomplete evidence supporting the synthesis of Tyr and Phe. However, the branch via anthranilate to Trp was absent. The His and Val/Leu/Ile pathways were also absent. Incomplete pathways were present for Thr, Met, Arg and Lys. A complete understanding of the biosynthetic capacity of *P. brassicae* will require further investigation, but it is clear that this obligate biotroph has lost the capacity to synthesise a number of amino acids and thus must acquire them from the host. The predicted transcriptome contained 10 putative amino acid transporters (Additional file 16: Table S8).

Inspection of the *P. brassicae* genome showed a complete glycolytic and TCA cycle, indicating a capacity for the utilization of carbohydrate from the host for energy production. However, unlike many biotrophic pathogens (including *P. infestans*), there was no evidence for extracellular invertase genes that would degrade host apoplastic sucrose. Therefore *P. brassicae* is likely to take up sugars directly. The genome contained 37 ABC transporters, some of which may be involved in nutrient acquisition. PbPT3Sc00087_Am_0.68_1 showed homology (28 %, *E*-value = 6e-11) to a SWEET sugar permease from *Ascaris suum*, PbPT3Sc00016_A_10.333_1 was predicted to encode a sucrose proton transporter, whilst PbPT3Sc00069_Am_1.146_1 and PbPT3Sc00030_A_2.294_1 encode putative monosaccharide transporters. The predicted sucrose transporters were expressed most strongly in developing galls (weeks 4–6 in *B. napus* and 26 DPI in *A. thaliana*) indicating that these genes may play a role in the development of sink strength during gall formation.

**Fig. 3:**
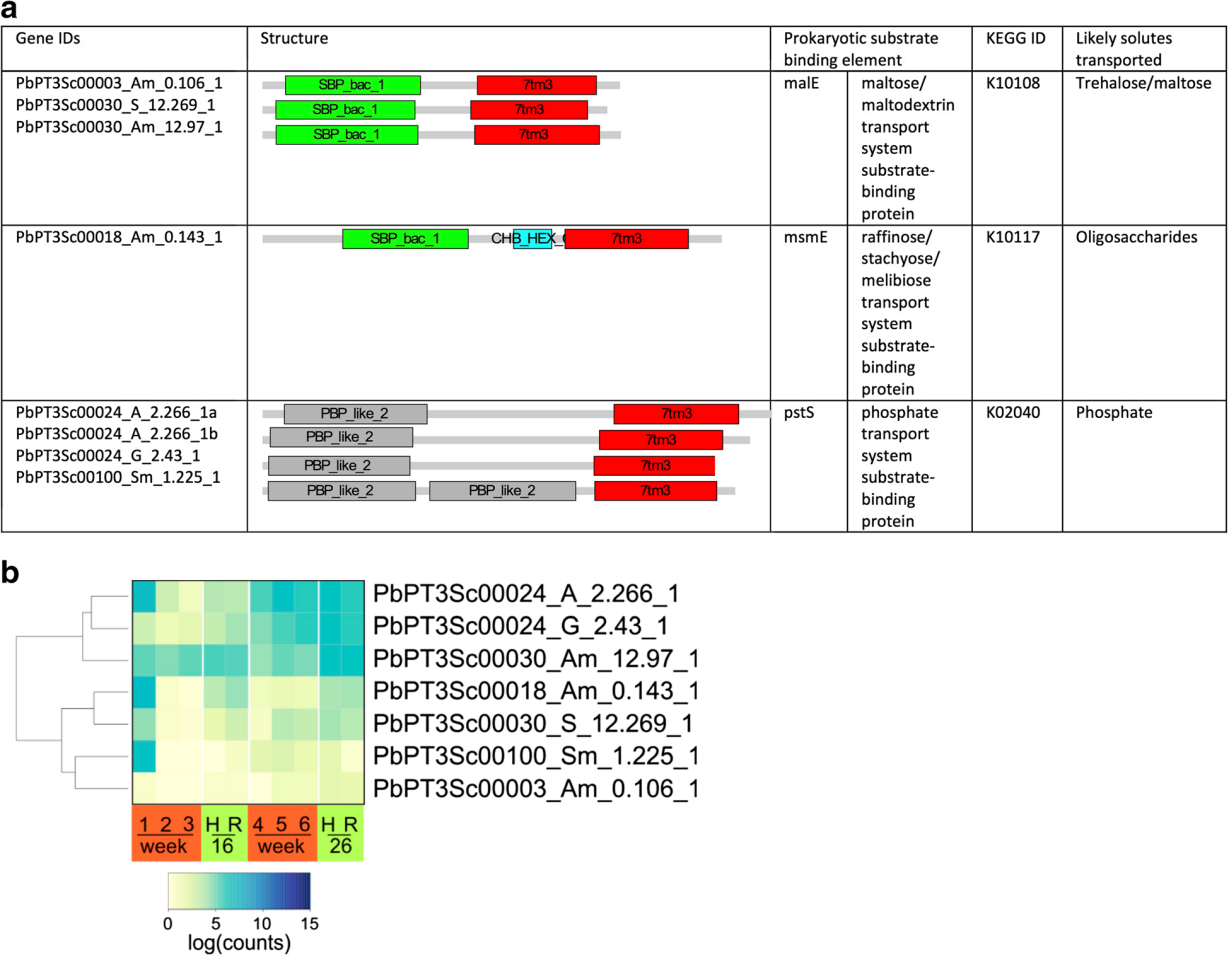
**a** Novel putative transporters in the *P. brassicae* genome fuse a eukaryotic 7tm3 motif with prokaryotic substrate binding domains. PFAM domains: SBP_bac_1 bacterial extracellular solute-binding protein; CHB_HEX N terminal domain of chitobiases, PBP_like_2 periplasmic binding protein. Homologies of these domains to known prokaryotic substrate binding elements are listed. **b** Expression in *P. brassicae*-infected *B. napus* (*red boxes*: wk1–6) and *A. thaliana* (*green boxes*) (16 and 26 DPI hypocotyls (H) and roots (R))

During the annotation process, in which predicted gene sequences were compared with known proteins in the KEGG, a series of matches were found against proteins with prokaryotic substrate binding domains. Inspection of these sequences found that these encoded a hitherto unknown class of eukaryotic transporters in which a prokaryotic substrate binding domain is fused to a 7 alpha-helical transmembrane domain. The structure, predicted solutes transported and expression pattern are shown in Fig. 3. No such fusion proteins were found in any other eukaryotic genome suggesting that this class of transporter is unique to *P. brassicae.* The gene sequences are present within the e3 genome but either not annotated as genes or annotated as two separate genes. However, our transcriptome analysis shows clearly that these are single coding regions in each case. Although characterisation of the substrates transported by these proteins requires experimental confirmation, sequence homology suggests that PbPT3Sc00003_Am_0.106_1, PbPT3Sc00030_S_12.269_1, PbPT3Sc00030_Am_12.97_1 and PbPT3Sc00018_Am_0.143_1 encode sugar (trehalose, maltose or oligosaccharide) transporters, whilst PbPT3Sc00024_A_2.266_1a, PbPT3Sc00024_A_2.266_1b, PbPT3Sc00024_G_2.43_1 and PbPT3Sc00100_Sm_1.225_1 encode phosphate transporters. The majority of these transporters were expressed early in infection and during gall development, suggesting they play roles in nutrient acquisition at these key developmental stages.

In common with many pathogens, *P. brassicae* has been shown to synthesize trehalose. The Pb3 genome contained 3 genes encoding trehalose phosphate synthase (TPS) (PbPT3Sc00016_A_8.299_1, PbPT3Sc00018_Sm_4.147_1, PbPT3Sc00059_A_1.289_1) and single genes encoding trehalose phosphate phosphatase (PbPT3Sc00030_A_6.312_1) and trehalase (PbPT3Sc00031_A_12.282_1) in agreement with [21]. The TPS gene PbPT3Sc00059_A_1.289_1 matched the fragment isolated by [36] and the corresponding complete gene reported elsewhere [37].

### Regulation of growth hormones during *P. brassicae* infection

The plant hormones cytokinin and auxin have long been implicated in Pb infection and symptom development. Current models favour the involvement of cytokinin in early stages of gall formation with auxin up-regulated at later stages. This model was confirmed by the expression profile of genes, related to plant hormone pathways, during the course of infection of *B. napus* with Pb3 (Additional file 12: Figure S7). The picture, however, remains complex [9]. Many gall-forming pathogens have the ability to synthesize plant growth regulators, or related substances and modulate the phytohormone response of the host. For example, *Rhodococcus fascians* and *Agrobacterium tumefaciens* produce auxin and cytokinins to modulate host plant physiology [38, 39]. There is also evidence that Pb can synthesize cytokinin albeit in limit amounts [40, 41].

Cytokinins are derived from adenine and are classified as isoprenoid or aromatic CKs based on their side chain. Common isoprenoid CKs in plants are trans-zeatin (tZ), cis-zeatin (cZ) and isopentenyl adenine (iP). CK biosynthesis in plants occurs via two routes. The synthesis of iP and tZ is catalyzed by an isopentenyl transferase (IPT) that preferentially uses ADP or ATP as the isoprenoid acceptor from dimethylallylpyrophosphate (DMAPP). Alternatively, IPTs that preferentially use tRNA as a substrate lead to the synthesis of cZ. In *A. thaliana*, adenine IPTs are the most important with iP and tZ the dominant active CKs – the cZ content is low (and cZ has much lower CK activity). We searched the predicted proteome of Pb3 for genes related to cytokinin biosynthesis using the KEGG Automatic Annotation Server (KAAS). Two genes were identified with close homology to IPT, PbPT3Sc00024_Sm_2.181_1 and PbPT3Sc00040_Am_6.202_1. Although these enzymes were predicted to be tRNA IPTs associated with cZ production, identification of substrate specificity via sequence alignment is unreliable and these may be adenine IPTs. We favour this latter view as [40] found that isolated plasmodia supplied with adenine had the capacity to synthesise tZ and infected *Arabidopsis* plants do not contain significant amounts of *cZ* [41]. The genome also contained all of the genes required for the synthesis of isopentenyl pyrophosphate from acetyl coA via the mevalonate pathway and its conversion to DMAPP by isopentenyl-diphosphate_delta-isomerase (PbPT3Sc00058_Am_5.127_1) (Additional file 17: Figure S9).

A predicted protein (PbPT3Sc00039_A_3.298_1) with strong homology to an indole-3-acetic acid amido synthetase that synthesizes auxin-amino acid conjugates was also present in the genome. In plants this process is involved in auxin homeostasis and clubroot has been suggested to act as a sink for auxin [9].

## Conclusion

Studies into the genetics and biology of *P. brassicae* have proven to be difficult because this pathogen is a soil-borne obligate biotroph completing its life cycle inside the host plant cell. The draft genome sequences presented here, along with the re-sequencing data of multiple pathotypes, adds to the previously published genome sequence [21] to provide a set of foundational resources that will partly overcome these limitations. *P. brassicae* shares many features previously reported for other biotrophic pathogens, namely a reduction in plant cell wall degrading enzymes, a loss of genes for uptake and assimilation of inorganic nitrogen and sulfate, and a loss of enzymes for thiamine biosynthesis and reduction of genes encoding for the production of toxins. These are common features identified in several biotrophic fungal and oomycete plant pathogens [31]. What distinguishes the *P. brassicae* genome is the small size and scarcity of repeats in comparison with most of other obligate biotrophs such as rust and powdery mildew fungi and the downy mildew oomycete *Hyaloperonospora arabidopsidis* [31]. Transposable and other repetitive elements are factors that contribute to genome expansion, evolution and possible diversification of effector genes in biotrophic fungi. Another unique feature of the *P. brassicae* genome is the reduction (and in some occasions overlapping UTRs) of intergenic distances. This resembles the reduction of intergenic intervals in microsporidia, another group of obligate intracellular parasites in which the reduction of gene space has forced promoters and terminators into adjacent genes and consequently the presence of mRNAs that encode sequences from more than one gene [24].

Comparison of the SNP profile of Pb3 with four other Canadian pathotypes showed a clear difference between Pb3 and Pb6 while the SNP profile for Pb2, Pb5 and Pb8 were very similar. Interestingly this SNP-based grouping correlated with host specificity of these isolates. Despite the marked difference between the genome wide SNP profile of Pb3 and Pb6, these two pathotypes were very similar with respect to genome structure and their effector profile.

As a biotrophic pathogen *P. brassicae* relies on host plant carbon sources for growth. Among the Pb3 predicted transporters were a monosaccharide, a sucrose and several major facilitator superfamily (MSF) transporters that transport different compounds including simple sugars. We identified a novel group of transporters among the predicted proteins of Pb3 with substrate binding motifs only found in prokaryotic ABC transporters [42]. Seven proteins with these bacterial transporter motifs had 7 transmembrane sweet-taste receptor of 3 GCPR (7tm3) joined with either a bacterial extracellular solute-binding protein (SBP) or a bacterial periplasmic-binding (PBP) domain. Bacterial SBP motifs are essential for transmembrane transport irrespective of solute concentration, as they restrict substrate diffusion away from the cell and improve transporter affinity [43, 44]. The evolutionary origin of these transporters remains to be established but they are likely to represent examples of horizontal gene transfer from prokaryotes to eukaryotes which, whilst relatively rare, has been documented in other protist genomes [45]. Schwelm et al. [21] also reported that a number of *P. brassicae* GH18 chitinases were closely related to bacterial chitinases which have not been reported in other eukaryotes before.

Plant biotrophic pathogens are efficient in evading host recognition and suppressing plant defence through secretion of effector proteins. We identified 221 small secreted proteins among the predicted Pb3 proteins of which 60 % (132 SSPs) showed no homology to the NCBI nr database. Twenty-eight SSPs that were differentially expressed during infection consisted of proteins with chitin binding domains, proteinase activity and peptidase inhibitors. Salicylic acid (SA) is the main plant hormone for activating defence against biotroph pathogens. We identified a putative Pb3 effector with homology to plant protein BSMT that function as SA methyl transferase. *In planta* BSMT generates MeSA at the site of pathogen infection. MeSA acts as a SAR signal in distal leaves of infected plants. A BSMT from *P. brassicae* was recently reported by Ludwig-Müller and colleagues [30]. Here we also showed the function of this gene in induction of SAR and that it was differentially expressed during infection. One possible role for *P. brassicae* BSMT is to dampen the effect of SA in local tissue by converting it to MeSA with the potential benefit of protecting above-ground parts of the plant from other pathogens, and thus sustaining the life of the host plant that is required for obligate biotrophic pathogens. SA mediated plant defence is mainly described for foliar plant pathogens and there is little information about involvement of the SA dependent defence pathway in roots. An example of the possible involvement of SA against root colonising pathogens is the induction of a SA marker gene encoding pathogenesis related protein 1 (PR-1) in barley roots during challenge with *Bipolaris sorokiniana*, a root pathogen of barley [46]. Similarly in a recent paper Lemarié et al., [47] reported that in *Arabidopsis* plants with partial resistance to clubroot SA was induced. Two *B. napus* orthologues of *Arabidopsis PR1* (At2g14610, [48]) were expressed in *B. napus* roots infected with Pb3 but only one of these orthologues was differentially expressed. Expression of this gene started at week 3 and then increased at weeks 4 and was significantly up-regulated at weeks 5 and 6 (7-fold at week 5 and 12-fold at week 6). This pattern of *PR1* gene expression suggests the suppression of the SA pathway at earlier stages of infection, as was also supported by our HORMONOMETER analysis.

## Methods

### *P. brassicae* isolates and plant inoculation

The *P. brassicae* single-spore isolates SACAN-ss3 (classified as Pb2), SACAN-ss1 (Pb3), ORCA-ss4 (Pb5), AbotJE-ss1 (Pb6) and ORCA-ss2 (Pb8) [18] were used for genome sequencing and transcriptomic analysis during *B. napus* infection. The isolates were maintained as resting spores in mature root galls of the universally susceptible host Chinese cabbage (*B. rapa* var. *pekinensis*) ‘Granaat’ at −80 °C. *B. napus* cv DH 112075 was derived from a cross between *B. napus* cultivars Cresor and Westar. Extraction of the resting spores and host inoculation were carried out as described by Strelkov et al. [20] for frozen root tissue. The inoculated seedlings were grown in Sunshine #4 Mix/LA7 potting medium (SunGro Horticulture) and kept in a greenhouse at 20 ± 2 °C with a 16 h photoperiod (light intensity of approximately 300 μmol/m^2^/s). Galled roots were harvested 6 weeks after inoculation for the collection of resting spores. Resting spores to be used for the isolation of nucleic acids were purified further by using a continuous gradient of HS-40 colloidal silica (LUDOX, 40 wt% suspension in water; Sigma-Aldrich Canada Ltd.) as per the protocol of Castelbury et al. [49].

Additional transcriptomic analysis was performed on *A. thaliana* Col-0 inoculated with a Welsh pathoype of *P. brassicae* as described in [13]. This isolate was maintained on *B. rapa* var. *pekenensis* ‘Wong-Bok’ and plants were inoculated with 1 ml of 10^6^ spores ml^−1^ 14 days after germination. Plants material (hypocotyls and the upper 1 cm of root) were harvested 16 and 26 days post inoculation.

### DNA and RNA extraction

Purified mature spores of *P. brassicae* were collected from macerated clubs formed on roots of susceptible plants as described by Cao et al. [50]. Tissue (100–200 mg) was ground in liquid nitrogen using a mortar and pestle and then homogenized in Cell Lysis Solution-VF or CLS-Y using the Lysing Matrix A tube supplied with the kit and the single tube Mini-Beadbeater from Biospec Products. Homogenization was performed in 3 × 1 min cycles at 4800 rpm with 1 min of cooling on ice in between. DNA was eluted, treated with RNAse A followed by Phenol:Chloroform:Isoamyl alcohol (P:C:I) extraction and C:I extraction, precipitated and re-suspended in 10mMTris-HCl pH8.5. RNA was extracted from the roots of infected plants. Tissues were ground in liquid nitrogen, and RNA was extracted with TRIzol and Ambion PureLink RNA Mini kit (Cat# 12183-019A, Life Technologies, USA) according to the manufacturer’s protocol. RNA quantity and quality were determined by Qubit (Life Technologies, USA) and Bioanalyzer (Agilent Technologies Inc. USA), respectively. RNA concentration for different samples was in the range of 57–100 ng μl^−1^.

### Genome sequencing and annotation

DNA from purified spores of Pb3 and Pb6 was used for shotgun and mate-paired sequencing using Roche 454 Titanium chemistry following the manufacturer’s protocol (Roche, USA). DNA sequencing was carried out at the Applied Genomics and Analytical Technologies Dept., National Research Council of Canada. Raw sequencing reads were mapped to the genome of *B. napus* cv. DH12075 (Parkin et al. unpublished) or *B. rapa* cv. Chiifu [51] to remove the possible plant DNA sequences in the Pb3 and Pb6 samples, respectively. *P. brassicae* genomes were assembled using Newbler assembly software (v. 2.3; Roche, USA). RepeatMasker and RepeatModeler software [52] were used to analyse the repeats within the *P. brassicae* genomes.

Genome annotation was conducted using the *ab initio* gene prediction software Maker (v. 2.22) [53]. The software was configured to use RNA sequence reads from infected roots and assembled 454 RNA reads generated from *P. brassicae* spores as well as SwissProt proteins as evidence. The gene prediction tools SNAP and Augustus trained on *P. bassicae* were used by Maker in an iterative fashion to predict *P. brassicae* genes.

Putative transcript functions and GO analysis were performed using BLAST2GO [25]. The entire annotation process was conducted for Pb3 and Pb6 independently, using only data from the one pathotype (i.e. no inter-pathotype data were used for training SNAP and Augustus).

A pipeline that is commonly used to predict secreted proteins from filamentous fungi [54] was adapted for prediction of Pb3 secreted proteins. The criteria included in the search were presence of an N-terminal signal peptide, lack of transmembrane and membrane anchoring domains, and absence of subcellular localisation signals using SignalP (v4.1), TMHMM (v2), PredGPI and TargetP (v1.1) software, respectively [55–58]. The fasta file of mature proteins generated by SignalP was used as input file for TMHMM. Proteins without a transmebmbrane helix that also lacked GPI and defined as secreted (S) by TargetP (no RC threshold included) were considered as secreted proteins. SSPs with an RXLR motif were identified based on the criteria described previously [59, 60] using the RXLR motif finding tool provided by the Galaxy software [61–64].

### RNA-sequencing and transcriptomic analysis

RNASeq was performed at the Applied Genomics and Analytical Technologies Dept., National Research Council of Canada. Using the Illumina Hi-Seq 2500 system a total of 1,075,036,260 sequence reads (about 89.6 million reads per time course) were generated from paired-end RNA-seq libraries from *B. napus* root tissues 1, 2, 3, 4, 5 and 6 weeks after inoculation with *P. brassicae,* as well as libraries mock inoculated with water. All generated sequences were processed using the CASAVA algorithm prior to being aligned to the *P. brassicae* genome sequence using the RNA-Seq analysis application from CLC Genomics workbench (v. 7.0.4), where 89,317,981 sequence reads were aligned to the *P. brassicae* genome. Under the assumption that no *P. brassicae* sequences should be present in the water-inoculated samples, the sequences detected in the water-inoculated libraries were subtracted from the values obtained in the *P. brassicae* inoculated libraries.

For the *A. thaliana* transcriptome analysis, paired-end RNA-seq libraries were prepared from the upper 1 cm root or hypocotyls 16 and 26 days after inoculation. Approximately 25 million reads were obtained per sample. Three independent biological replicates were analysed for each time point with the appropriate uninfected controls. Paired end reads were mapped using Stampy [65] to the *A. thaliana* genome TAIR10 (https://www.arabidopsis.org/) and *P. brassicae* Pb3 genome. More than 99 % of the reads mapped to one genome or the other.

Reads matching *P. brassicae* transcripts were counted using HTSeq [66] with the default ‘union’ method. Differential gene expression, corrected for false discovery using the Benjamini-Hochberg method, was identified using DeSEQ2 [67]. For the *B. napus* data comparisons were made between each week and for the *A. thaliana* data between each time point (16 and 26 DPI) in a tissue type (root or hypocotyl). For the production of heatmaps, the output of the DESeq2 analysis was subject to regular log normalisation and heatmaps drawn using the R package heatmap.2.

### Hormonal signature analysis

The putative *A. thaliana* orthologues of differentially expressed genes (DEG) at different time point comparisons in *B. napus* were imported to HORMONOMETER [29] to identify transcriptional similarities between DEG and *A. thaliana* genes associated with various plant hormones.

### Detection of SNPs and indels

Reads from each pathotype were aligned independently to the reference genome using CLC Genomics Workbench (v. 7.0.4). For each pathotype, a Binary Alignment Map (BAM) file was exported from the CLC Genomics Workbench. SNPs in all genotypes were called using the Mpileup command from Samtools (v. 0.1.18) to produce a Variant Call Format (VCF) file. The VCF file was filtered to remove positions where the variant allele frequency was 100 %. Additionally, if a single pathotype had a heterozygous genotype call, the position was removed. Due to the presence of homopolymer repeat errors in the 454 genome sequence, indels were found to be unreliable and were excluded from further analysis. The filtered VCF file was then processed using a custom Perl script to determine the number of haplotypes based on identical SNP patterns. Since the scaffolds of the reference genome are unordered, haplotype blocks were stopped when the end of a scaffold was reached. The recently published *P. brassicae* e3 isolate genome sequence [21] was aligned to the reference genome Pb3 using the Nucmer application within MUMmer (v 3.23) [52]. SNPs were called from the aligned genome using the MUMmer application “show-snps”. These SNPs were compared with the SNPs identified from the other pathotypes to define common SNPs.

### Availability of data

Genome sequence of *P. brassicae* pathotypes 2, 3, 5, 6 and 8 has been deposited into the public domain in GenBank (Submission # 1899024).

## Additional files

Additional file 1: Figure S1. Reaction of the universally susceptible Chinese cabbage (*Brassica rapa* var. *pekinensis*) ‘Granaat’ and the differential hosts of Williams [16] to inoculation with selected pathotypes of *Plasmodiophora brassicae*. (A) Reaction of *Brassica* hosts to pathotype 3 of *P. brassicae*, from left: ‘Granaat’, *Brassica oleracea* var. *capitata* ‘Jersey Queen’, *B. oleracea* var. *capitata* ‘Badger Shipper’, *B. napus* var. *napobrassica* ‘Wilhemsburger’, and *B. napus* var. *napobrassica* ‘Laurentian’. Note extensive root galling (susceptible reaction) of ‘Granaat’, ‘Jersey Queen’ and ‘Laurentian’. (B) Reaction of ‘Laurentian’ to inoculation with pathotype 6 of *P. brassicae*. Note the absence of root galls. The reaction of the other hosts is the same as for pathotype 3. (PDF 741 kb) Additional file 2: Table S1. Assembly statistics for *P. brassicae* pathotypes 3 and 6 (Pb3, Pb6). (DOCX 13 kb) Additional file 3: Figure S2. Intergenic distances between *P. brassicae* genes. The intergenic distances at the 5′ and 3′ end of each transcript were calculated for all genes in the Pb3 genome except those at the very ends of a scaffold. These were sorted into bins and plotted as a heat map with the number of genes in each bin colour coded. (PNG 1524 kb) Additional file 4: Figure S3. A selected region of the *P. brassicae* genome showing (A) very small intergenic regions and (B) overlapping transcripts from adjacent genes. (PDF 340 kb) Additional file 5: Figure S4. The *P. brassicae* pathotype 6 shows extensive synteny with pathotype 3 with rare examples of small inversions detected between Pb3 and Pb6. Images shows Pb3 scaffold 59 alignment with Pb6 scaffolds 255 and 179. (PNG 215 kb) Additional file 6: Table S2. Position of SNPs idnetified among different pathotypes of *P. brassicae*, using pathotype 3 as reference. (XLSX 7838 kb) Additional file 7: Table S3. Gall formation of *P. brassicae* pathotypes on (A) selected *B. napus* and *B. olereacea* hosts and (B) selected European Clubroot Differential (ECD) lines and *B. napus* hosts. Note the distinct virulence pattern of AbotJE-ss1 (Williams’ pathotype 6). (DOCX 15 kb) Additional file 8: Figure S5. Graphical representation of the 59 SNP haplotypes discovered across 5 pathotypes (The e3 genome was reported by Schwlem et al. [21]). Pathotypes alleles are coded in green or red for the reference (Pb3) or alternate alleles, respectively. Clustering was performed using the default dist fuction of the statistics package R. (PDF 172 kb) Additional file 9: Table S4.
*P. brassicae* pathotype 3 predicted secreted proteins. (XLSX 18 kb) Additional file 10: Table S5. A list of small secreted proteins (SSPs) of *P. brassicae* that contains potential RXLR motifs. Names displayed in bold are SSPs where the position of RXLR and EER motifs match the model described by Bhattacharjee et al. [59] and Win et al. [60]. (XLSX 11 kb) Additional file 11: Figure S6. Gene Ontology terms that are enriched in the test group compared with the whole genome using Fisher’s Exact Test with Multiple Testing Correction of FDR (Benjamini and Hochberg). The test group were genes that are (A) down-regulated or (B) up-regulated in *A. thaliana* 16 DPI tissue compared to the 26 DPI sample. The reference set was all Pb3 *P. brassicae* genes. Only genes that showed differential expression in both root and hypocotyl tissue are included. (PNG 437 kb) Additional file 12: Figure S7. Hormone-responsive gene expression profile of *B. napus* roots in response to *P. brassicae* infection. The HORMONOMETER software was used to compare gene expression data of the query (*B. napus* during infection with *P. brassicae*) with the expression profile of *A. thaliana* genes in response to various hormones. Root samples were taken at weekly intervals after infection starting at week 1 (W1) until week (W6). Red color represents a positive correlation and blue color represents a negative correlation between the gene expression of the query and the *A. thaliana* transcripts in response to hormones. (PNG 935 kb) Additional file 13: Figure S8. Transient expression of PbPT3Sc00026_A_1.308_1, a benzoic acid/salicylic acid methyltransferase (BSMT) leads to a reduction in bacterial cell numbers and lesion size in *N. tabacum* infiltrated with *Pseudeomonas syringae* pv. *tabaci* in distal leaves. Expression of GFP was used as a control. (PPTX 266 kb) Additional file 14: Table S6. A list of predicted *P. brassicae* pathotype carbohydrate-active enzymes. (DOCX 22 kb) Additional file 15: Table S7. Biosynthetic pathways for amino acids and their relative completeness in the *P. brassicae* genome. (DOCX 14 kb) Additional file 16: Table S8. Putative amino acid transporters. (DOCX 14 kb) Additional file 17: Figure S9. The terpenoid biosynthesis pathway with enzymes encoded in the *P. brassicae* genome coloured green. (PNG 823 kb)
