# Supplementary figures and images for: The compact genome of the plant pathogen Plasmodiophora brassicae is adapted to intracellular interactions with host Brassica spp
Source: BMC Genomics. 2016 Mar 31;17:272. doi: 10.1186/s12864-016-2597-2 (PMC4815078; doi:10.1186/s12864-016-2597-2)

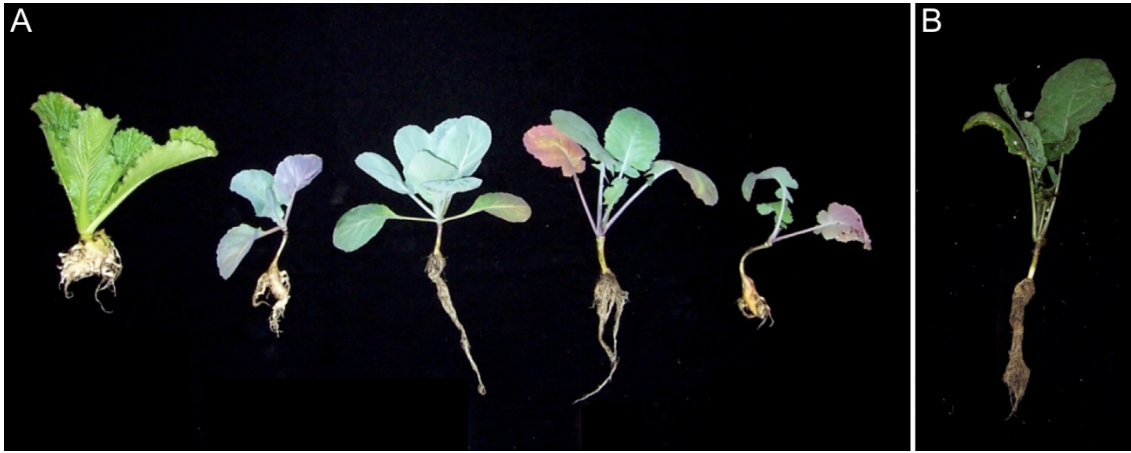

Supplement: Additional file 1: Figure S1. — Reaction of the universally susceptible Chinese cabbage (Brassica rapa var. pekinensis) ‘Granaat’ and the differential hosts of Williams [16] to inoculation with selected pathotypes of Plasmodiophora brassicae. (A) Reaction of Brassica hosts to pathotype 3 of P. brassicae, from left: ‘Granaat’, Brassica oleracea var. capitata ‘Jersey Queen’, B. oleracea var. capitata ‘Badger Shipper’, B. napus var. napobrassica ‘Wilhemsburger’, and B. napus var. napobrassica ‘Laurentian’. Note extensive root galling (susceptible reaction) of ‘Granaat’, ‘Jersey Queen’ and ‘Laurentian’. (B) Reaction of ‘Laurentian’ to inoculation with pathotype 6 of P. brassicae. Note the absence of root galls. The reaction of the other hosts is the same as for pathotype 3. (PDF 741 kb) [file 12864_2016_2597_MOESM1_ESM.pdf]

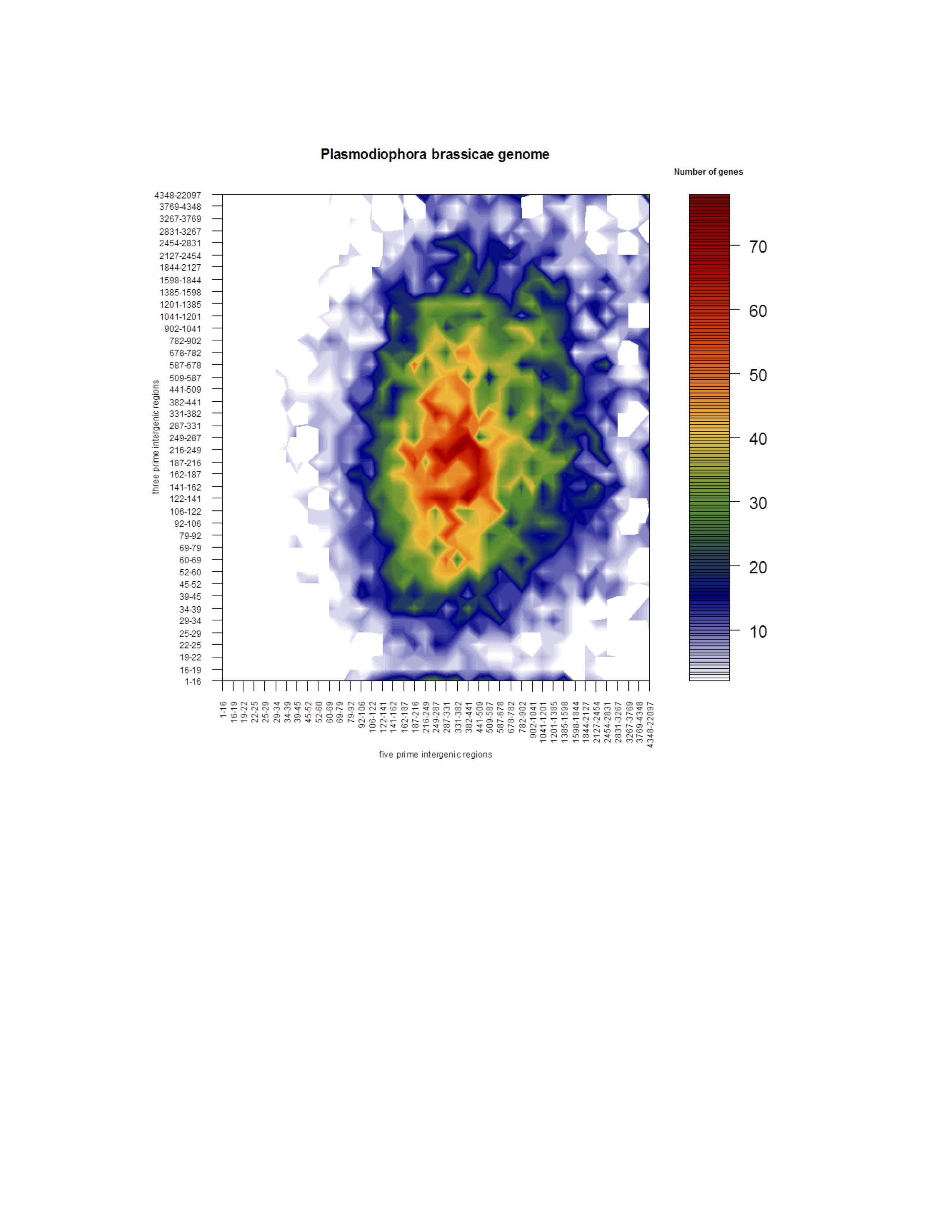

Supplement: Additional file 3: Figure S2. — Intergenic distances between P. brassicae genes. The intergenic distances at the 5′ and 3′ end of each transcript were calculated for all genes in the Pb3 genome except those at the very ends of a scaffold. These were sorted into bins and plotted as a heat map with the number of genes in each bin colour coded. (PNG 1524 kb) [file 12864_2016_2597_MOESM3_ESM.png]

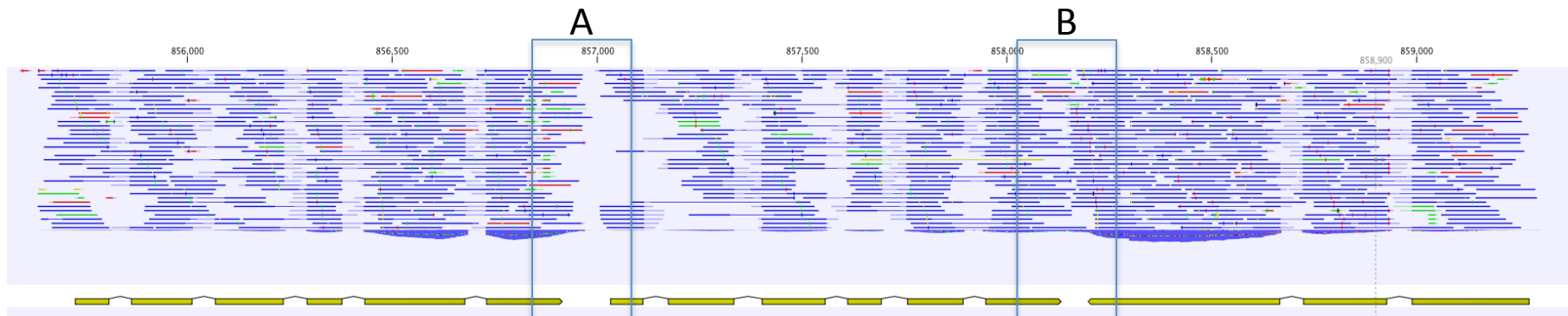

A

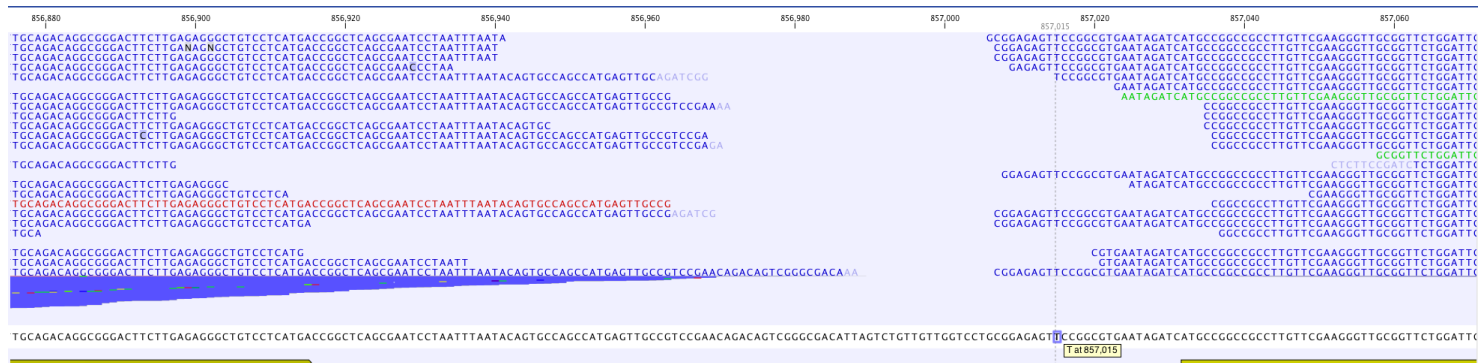

B

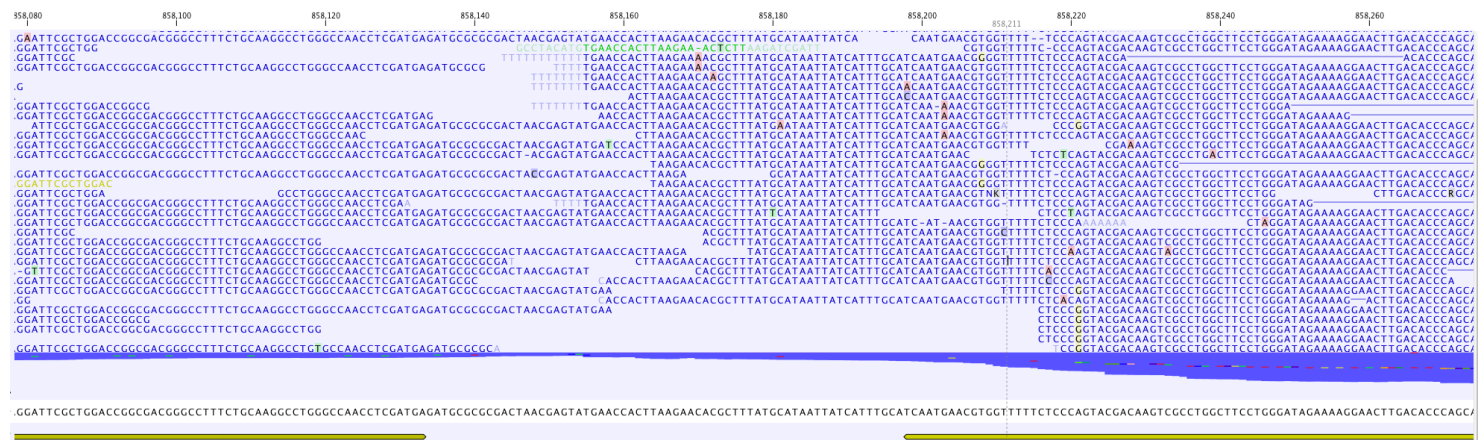

Supplement: Additional file 4: Figure S3. — A selected region of the P. brassicae genome showing (A) very small intergenic regions and (B) overlapping transcripts from adjacent genes. (PDF 340 kb) [file 12864_2016_2597_MOESM4_ESM.pdf]

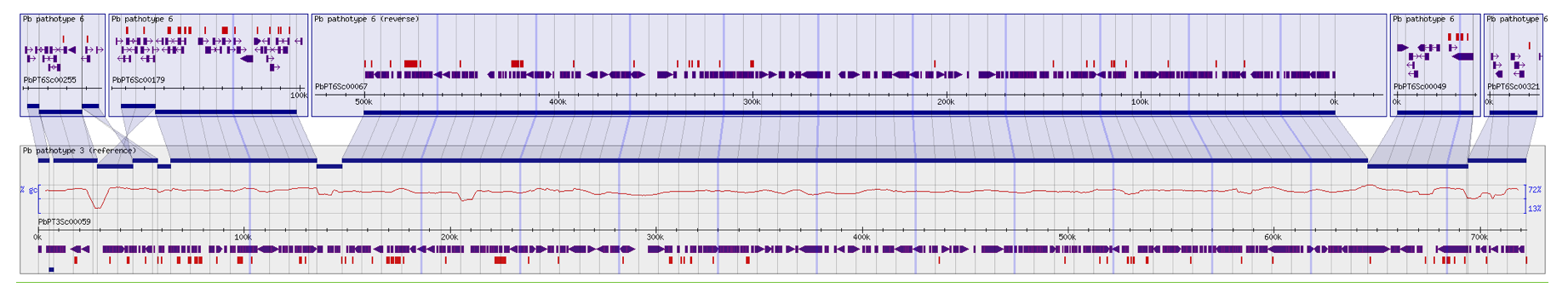

Supplement: Additional file 5: Figure S4. — The P. brassicae pathotype 6 shows extensive synteny with pathotype 3 with rare examples of small inversions detected between Pb3 and Pb6. Images shows Pb3 scaffold 59 alignment with Pb6 scaffolds 255 and 179. (PNG 215 kb) [file 12864_2016_2597_MOESM5_ESM.png]

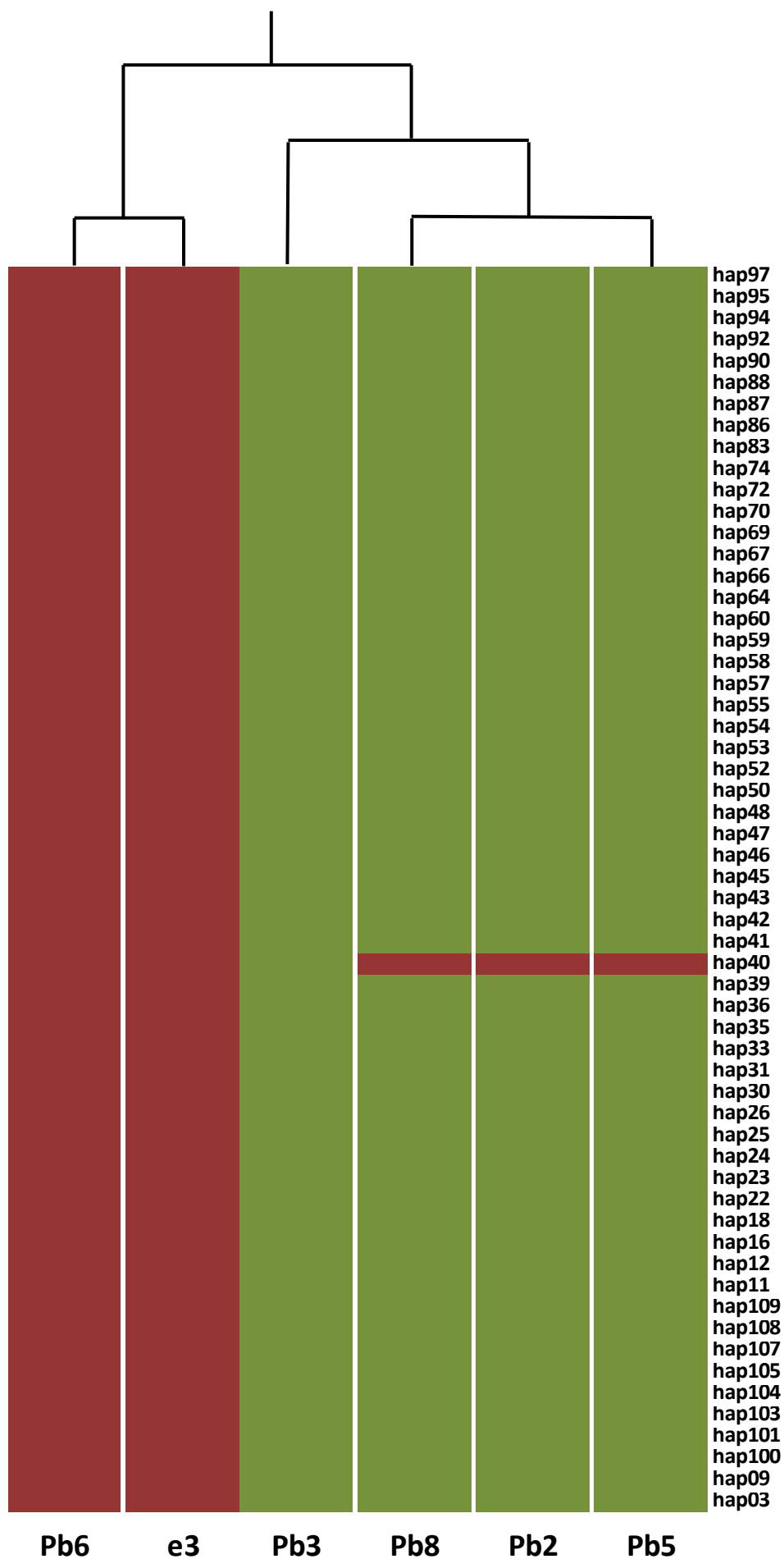

Supplement: Additional file 8: Figure S5. — Graphical representation of the 59 SNP haplotypes discovered across 5 pathotypes (The e3 genome was reported by Schwlem et al. [21]). Pathotypes alleles are coded in green or red for the reference (Pb3) or alternate alleles, respectively. Clustering was performed using the default dist fuction of the statistics package R. (PDF 172 kb) [file 12864_2016_2597_MOESM8_ESM.pdf]

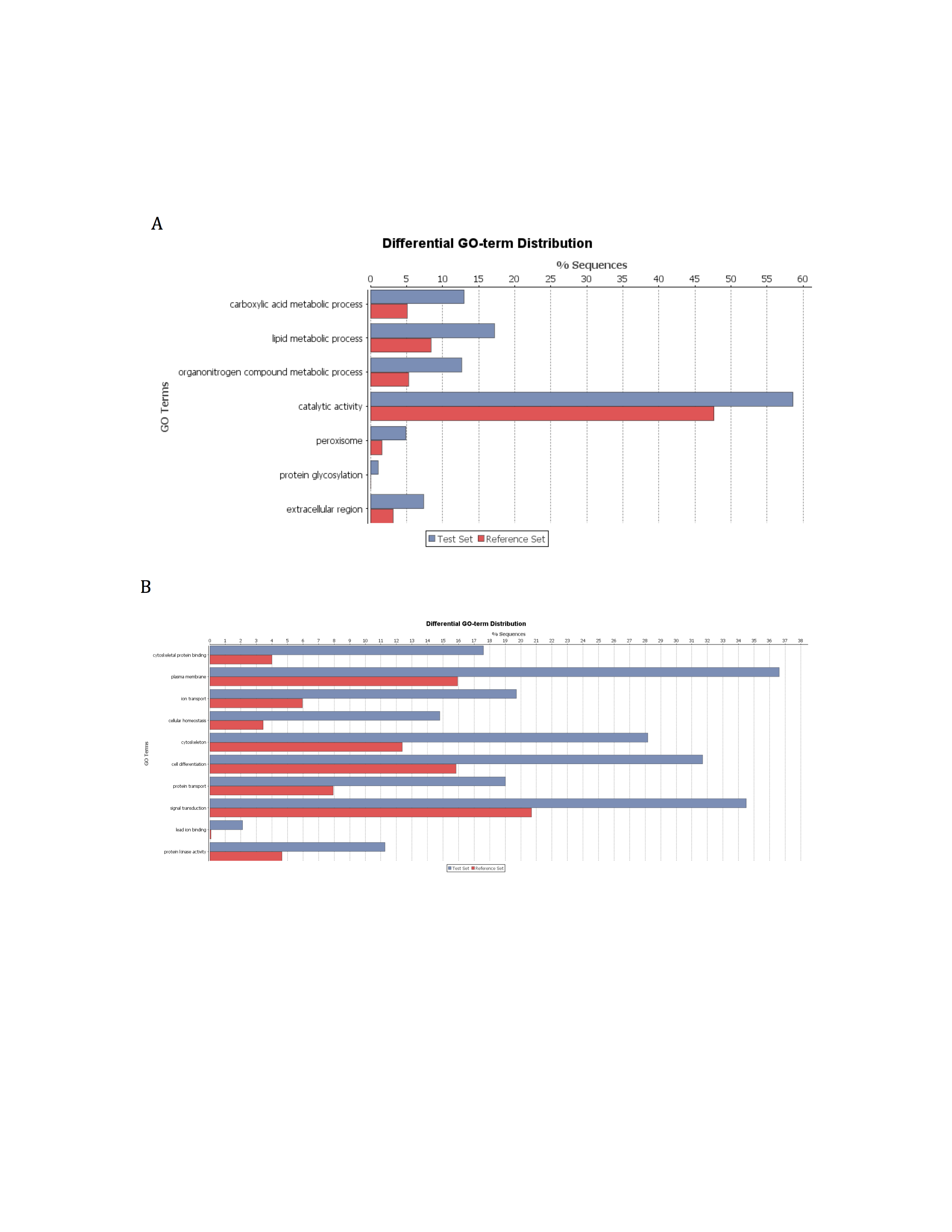

Supplement: Additional file 11: Figure S6. — Gene Ontology terms that are enriched in the test group compared with the whole genome using Fisher’s Exact Test with Multiple Testing Correction of FDR (Benjamini and Hochberg). The test group were genes that are (A) down-regulated or (B) up-regulated in A. thaliana 16 DPI tissue compared to the 26 DPI sample. The reference set was all Pb3 P. brassicae genes. Only genes that showed differential expression in both root and hypocotyl tissue are included. (PNG 437 kb) [file 12864_2016_2597_MOESM11_ESM.png]

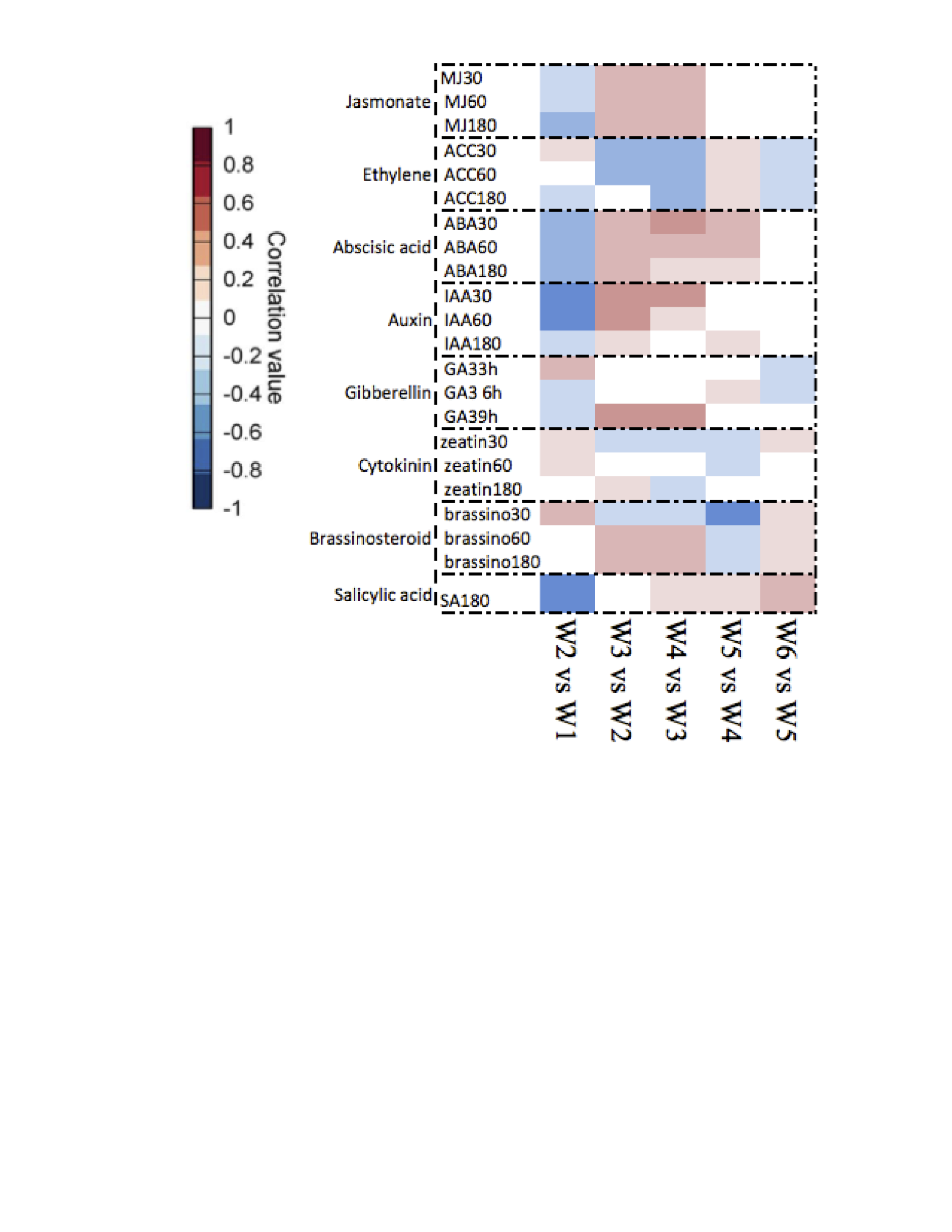

Supplement: Additional file 12: Figure S7. — Hormone-responsive gene expression profile of B. napus roots in response to P. brassicae infection. The HORMONOMETER software was used to compare gene expression data of the query (B. napus during infection with P. brassicae) with the expression profile of A. thaliana genes in response to various hormones. Root samples were taken at weekly intervals after infection starting at week 1 (W1) until week (W6). Red color represents a positive correlation and blue color represents a negative correlation between the gene expression of the query and the A. thaliana transcripts in response to hormones. (PNG 935 kb) [file 12864_2016_2597_MOESM12_ESM.png]

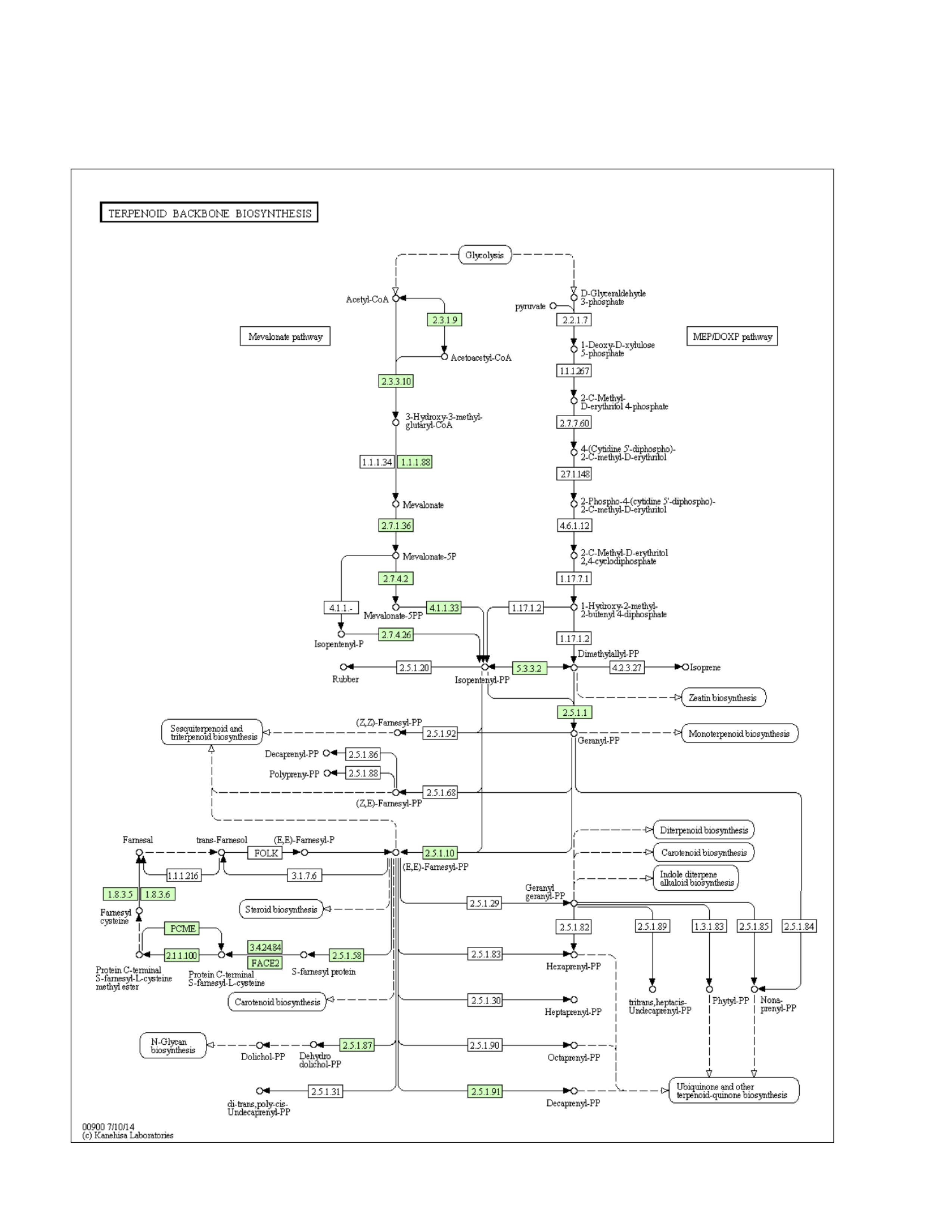

Supplement: Additional file 17: Figure S9. — The terpenoid biosynthesis pathway with enzymes encoded in the P. brassicae genome coloured green. (PNG 823 kb) [file 12864_2016_2597_MOESM17_ESM.png]
