# Supplementary material for: The compact genome of the plant pathogen Plasmodiophora brassicae is adapted to intracellular interactions with host Brassica spp
Source: BMC Genomics. 2016 Mar 31;17:272. doi: 10.1186/s12864-016-2597-2 (PMC4815078; doi:10.1186/s12864-016-2597-2)
Supplement: Additional file 2: Table S1. — Assembly statistics for P. brassicae pathotypes 3 and 6 (Pb3, Pb6). (DOCX 13 kb) [file 12864_2016_2597_MOESM2_ESM.docx]

**Additional file 2**

**Table S1**

|  | Pb3 | Pb6 |
| --- | --- | --- |
| Number of scaffolds | 107 | 356 |
| Number of bases (bp) | 24192107 | 24180791 |
| Average scaffold size (kbp) | 22.6 | 6.8 |
| N50 scaffold size (kbp) | 722.7 | 22.3 |
| largestScaffoldSize (Mbp) | 1.9 | 1.3 |
