# Supplementary material for: The compact genome of the plant pathogen Plasmodiophora brassicae is adapted to intracellular interactions with host Brassica spp
Source: BMC Genomics. 2016 Mar 31;17:272. doi: 10.1186/s12864-016-2597-2 (PMC4815078; doi:10.1186/s12864-016-2597-2)
Supplement: Additional file 7: Table S3. — Gall formation of P. brassicae pathotypes on (A) selected B. napus and B. olereacea hosts and (B) selected European Clubroot Differential (ECD) lines and B. napus hosts. Note the distinct virulence pattern of AbotJE-ss1 (Williams’ pathotype 6). (DOCX 15 kb) [file 12864_2016_2597_MOESM7_ESM.docx]

**Additional file 7**

**Table S3** Gall formation of *P. brassicae* pathotypes on (A) selected *B. napus* and *B. olereacea* hosts and (B) selected European Clubroot Differential (ECD) lines and *B. napus* hosts. Note the distinct virulence pattern of AbotJE-ss1 (Williams’ pathotype 6).

A

|  |  | | *Brassica napus* hosts | | | |  | *Brassica oleracea* hosts | |
| --- | --- | --- | --- | --- | --- | --- | --- | --- | --- |
| Isolate | | | Brutor | Laurentian | Nevin | Wilhemsburger |  | Badger Shipper | Jersey Queen |
| SACAN-ss3 | | Pb2 | + | + | + | - |  | + | + |
| SACAN-ss1 | | Pb3 | + | + | + | - |  | - | + |
| ORCA-ss4 | | Pb5 | + | - | - | - |  | - | + |
| AbotJE-ss1 | | Pb6 | + | - | - | - |  | - | + |
| ORCA-ss2 | | Pb8 | + | + | + | - |  | - | - |

B

| Isolate | | ECD05, ECD07, Brutor, 45H26, 46A76 | ECD02, ECD10, Mendel, 45H29 | ECD06, ECD09 | ECD08 | Westar |
| --- | --- | --- | --- | --- | --- | --- |
| SACAN-ss3 | Pb2 | + | - | + | + | + |
| SACAN-ss1 | Pb3 | + | - | + | + | + |
| ORCA-ss4 | Pb5 | + | - | + | + | + |
| AbotJF-ss1 | Pb6 | + | - | - | - | + |
| ORCA-ss2 | Pb8 | + | - | + | + | + |
