# Supplementary material for: The compact genome of the plant pathogen Plasmodiophora brassicae is adapted to intracellular interactions with host Brassica spp
Source: BMC Genomics. 2016 Mar 31;17:272. doi: 10.1186/s12864-016-2597-2 (PMC4815078; doi:10.1186/s12864-016-2597-2)
Supplement: Additional file 13: Figure S8. — Transient expression of PbPT3Sc00026_A_1.308_1, a benzoic acid/salicylic acid methyltransferase (BSMT) leads to a reduction in bacterial cell numbers and lesion size in N. tabacum infiltrated with Pseudeomonas syringae pv. tabaci in distal leaves. Expression of GFP was used as a control. (PPTX 266 kb) [file 12864_2016_2597_MOESM13_ESM.pptx]

## Slide 1
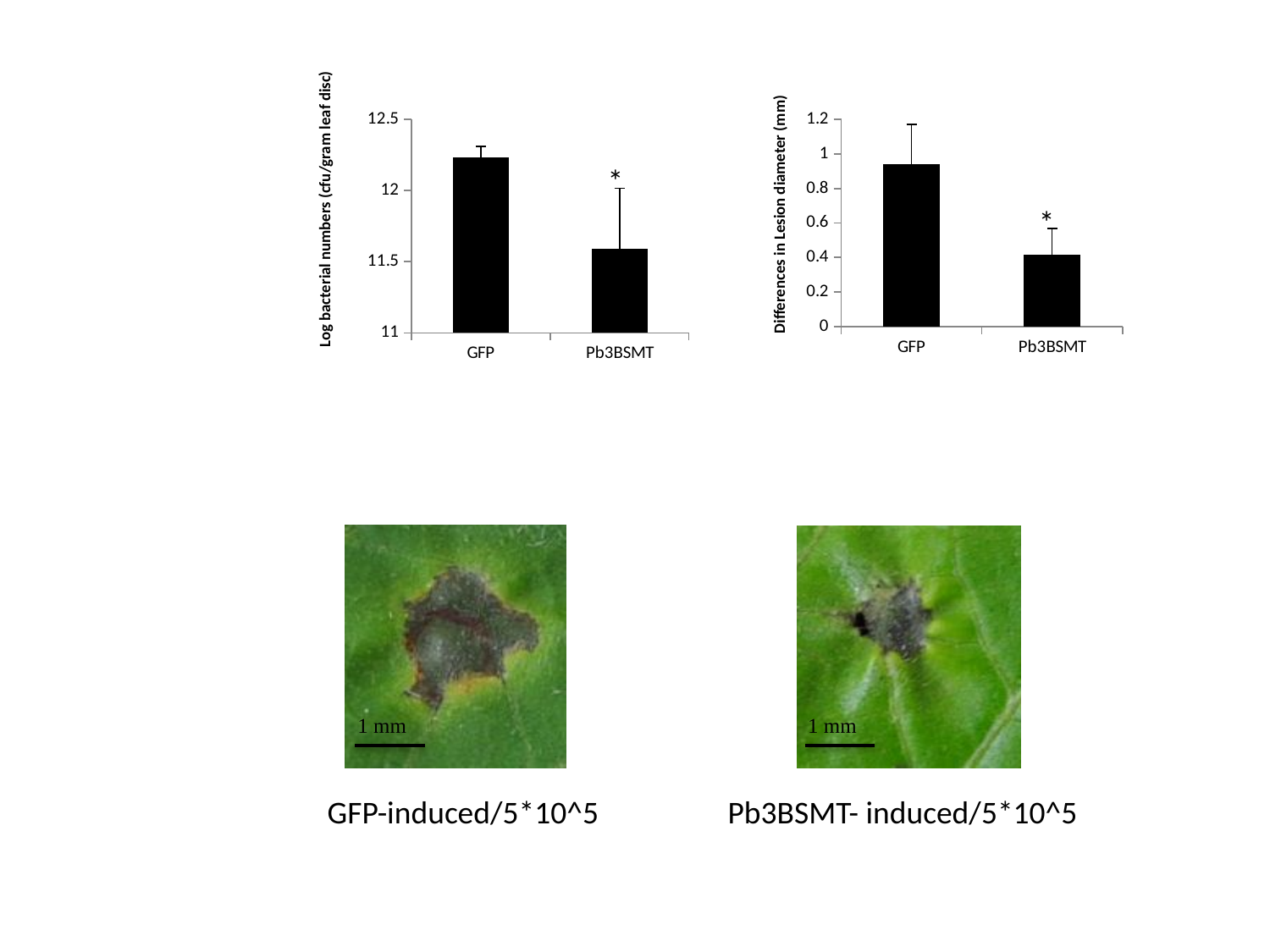

### Chart
| Category |
|---|
### Chart
| Category | |
|---|---|
| GFP | 0.939444444444443 |
| Pb3BSMT | 0.416666666666667 |
### Chart
| Category | |
|---|---|
| GFP | 12.23402671331127 |
| Pb3BSMT | 11.5921822154283 |Differences in Lesion diameter (mm)
*
Log bacterial numbers (cfu/gram leaf disc)
*
1 mm
1 mm
GFP-induced/5*10^5 Pb3BSMT- induced/5*10^5
