# Supplementary material for: The compact genome of the plant pathogen Plasmodiophora brassicae is adapted to intracellular interactions with host Brassica spp
Source: BMC Genomics. 2016 Mar 31;17:272. doi: 10.1186/s12864-016-2597-2 (PMC4815078; doi:10.1186/s12864-016-2597-2)
Supplement: Additional file 14: Table S6. — A list of predicted P. brassicae pathotype carbohydrate-active enzymes. (DOCX 22 kb) [file 12864_2016_2597_MOESM14_ESM.docx]

**Additional file 14**

Table S6 A list of predicted *Plasmodiophora brassicae* pathotype carbohydrate-active enzymes.

| **CAZY category** | **PB3 protein ID** | **# of genes** |
| --- | --- | --- |
| **Carbohydrate-binding modules** | | **38** |
| CBM2 | PbPT3Sc00095_A_0.137_1 | 1 |
| CBM13 | PbPT3Sc00030_Am_0.84_1 PbPT3Sc00030_Am_10.180_1 | 2 |
| CBM18 | PbPT3Sc00011_S_5.340_1 PbPT3Sc00016_Am_11.116_1 PbPT3Sc00023_A_7.270_1 PbPT3Sc00023_Sm_2.309_1 PbPT3Sc00030_Am_3.174_1 PbPT3Sc00030_Am_8.173_1 PbPT3Sc00031_Am_9.184_1 PbPT3Sc00033_Am_0.156_1 PbPT3Sc00039_Am_9.150_1 PbPT3Sc00039_Am_9.156_1 PbPT3Sc00048_S_5.266_1 PbPT3Sc00050_A_0.140_1 PbPT3Sc00057_Am_1.92_1 PbPT3Sc00072_Am_1.119_1 PbPT3Sc00086_S_1.318_1 PbPT3Sc00088_Sm_0.164_1 PbPT3Sc00108_Am_4.116_1 | 17 |
| CBM20 | PbPT3Sc00057_A_1.196_1 PbPT3Sc00108_G_2.90_1 | 2 |
| CBM21 | PbPT3Sc00058_A_1.187_1 PbPT3Sc00069_Sm_1.222_1 | 2 |
| CBM32 | PbPT3Sc00011_Am_11.116_1 PbPT3Sc00031_A_13.266_1 PbPT3Sc00039_Am_10.135_1 PbPT3Sc00040_Sm_5.224_1 PbPT3Sc00041_Am_3.127_1 PbPT3Sc00059_S_3.257_1 | 6 |
| CBM40 | PbPT3Sc00031_Sm_14.216_1 PbPT3Sc00059_Am_1.122_1 | 2 |
| CBM44 | PbPT3Sc00016_S_7.227_1 | 1 |
| CBM48 | PbPT3Sc00016_Am_0.107_1 | 1 |
| CBM50 | PbPT3Sc00031_A_16.107_1 | 1 |
| CBM53 | PbPT3Sc00031_Sm_5.269_1 | 1 |
| CBM56 | PbPT3Sc00047_Am_1.75_1 | 1 |
| CBM67 | PbPT3Sc00058_Am_7.136_1 | 1 |
| **Carbohydrate esterases** | | **58** |
| CE1 | PbPT3Sc00011_A_5.312_1  PbPT3Sc00011_Am_11.130_1  PbPT3Sc00011_S_0.146_1  PbPT3Sc00016_Am_10.155_1  PbPT3Sc00016_Am_4.32_1  PbPT3Sc00016_S_14.239_1  PbPT3Sc00023_A_5.281_1  PbPT3Sc00023_Am_2.194_1  PbPT3Sc00030_Am_8.187_1  PbPT3Sc00030_S_9.346_1  PbPT3Sc00030_Sm_11.186_1  PbPT3Sc00036_Am_4.130_1  PbPT3Sc00040_A_3.246_1  PbPT3Sc00058_A_3.289_1  PbPT3Sc00058_Am_8.147_1  PbPT3Sc00060_Am_1.116_1  PbPT3Sc00077_Am_0.146_1  PbPT3Sc00088_S_1.347_1 | 18 |
| CE4 | PbPT3Sc00011_Am_14.88_1 PbPT3Sc00011_Am_5.111_1 PbPT3Sc00023_A_7.270_1 PbPT3Sc00030_Am_10.157_1 PbPT3Sc00030_Am_11.76_1 PbPT3Sc00030_Am_8.173_1 PbPT3Sc00030_S_10.317_1 PbPT3Sc00031_Am_4.127_1 PbPT3Sc00031_Am_9.184_1 PbPT3Sc00039_A_4.359_1 PbPT3Sc00039_Am_3.164_1 PbPT3Sc00039_Am_7.103_1 PbPT3Sc00039_Sm_3.268_1 PbPT3Sc00050_A_0.140_1 PbPT3Sc00057_Am_1.92_1 PbPT3Sc00058_Am_7.137_1 PbPT3Sc00072_Am_3.125_1 PbPT3Sc00086_S_1.318_1 PbPT3Sc00088_Sm_0.164_1 PbPT3Sc00092_A_3.285_1 | 20 |
| CE7 | PbPT3Sc00072_A_3.259_1 PbPT3Sc00086_Am_0.82_1 | 2 |
| CE9 | PbPT3Sc00059_A_4.275_1 | 1 |
| CE10 | PbPT3Sc00011_Sm_1.219_1  PbPT3Sc00016_S_14.239_1  PbPT3Sc00031_Am_3.99_1  PbPT3Sc00036_A_3.329_1  PbPT3Sc00039_Am_9.137_1  PbPT3Sc00043_A_0.239_1  PbPT3Sc00048_A_1.306_1  PbPT3Sc00058_S_1.359_1  PbPT3Sc00060_S_0.250_1  PbPT3Sc00069_Am_2.128_1  PbPT3Sc00072_A_3.259_1  PbPT3Sc00086_A_3.125_1  PbPT3Sc00090_A_0.318_1  PbPT3Sc00097_Am_0.80_1  PbPT3Sc00108_A_3.289_1 | 15 |
| CE12 | PbPT3Sc00059_Sm_6.164_1 | 1 |
| CE14 | PbPT3Sc00030_Sm_0.178_1 | 1 |
| **Glycoside Hydrolases** | | **91** |
| GH2 | PbPT3Sc00009_S_2.272_1 | 1 |
| GH3 | PbPT3Sc00011_A_9.356_1 PbPT3Sc00031_Am_1.159_1 | 2 |
| GH5 | PbPT3Sc00011_G_13.72_1 PbPT3Sc00058_Am_0.91_1 PbPT3Sc00069_A_0.220_1 PbPT3Sc00077_Sm_0.172_1 PbPT3Sc00092_Am_6.121_1 PbPT3Sc00100_Am_1.168_1 | 6 |
| GH6 | PbPT3Sc00083_Am_0.81_1 | 1 |
| GH13 | PbPT3Sc00048_S_4.297_1 PbPT3Sc00100_Am_0.130_1 | 2 |
| GH15 | PbPT3Sc00094_A_0.228_1 |  |
| GH16 | PbPT3Sc00011_S_11.331_1 PbPT3Sc00011_S_3.261_1 PbPT3Sc00011_S_7.324_1 PbPT3Sc00016_Am_3.213_1 PbPT3Sc00016_S_11.249_1 PbPT3Sc00030_Am_8.134_1 PbPT3Sc00036_Am_5.156_1 PbPT3Sc00039_G_2.49_1 PbPT3Sc00039_Sm_4.261_1 PbPT3Sc00041_A_2.309_1 PbPT3Sc00041_Am_4.173_1 PbPT3Sc00048_Am_7.141_1 PbPT3Sc00059_G_5.105_1 | 13 |
| GH17 | PbPT3Sc00047_Sm_5.268_1 PbPT3Sc00047_Sm_5.273_1 PbPT3Sc00057_Am_2.152_1 | 3 |
| GH18 | PbPT3Sc00011_Am_1.174_1  PbPT3Sc00011_Sm_1.214_1  PbPT3Sc00016_A_11.234_1  PbPT3Sc00016_Am_14.130_1  PbPT3Sc00022_G_0.41_1  PbPT3Sc00030_S_6.225_1  PbPT3Sc00031_Am_17.143_1  PbPT3Sc00035_Am_0.2_1  PbPT3Sc00047_S_3.202_1  PbPT3Sc00047_S_3.250_1  PbPT3Sc00059_A_6.220_1  PbPT3Sc00069_Am_1.162_1  PbPT3Sc00072_Am_1.154_1  PbPT3Sc00083_A_0.201_1  PbPT3Sc00087_Sm_1.188_1  PbPT3Sc00100_Sm_1.238_1  PbPT3Sc00101_Am_0.65_1  PbPT3Sc00101_Am_0.77_1  PbPT3Sc00108_A_1.93_1 | 19 |
| GH19 | PbPT3Sc00030_Sm_8.270_1 PbPT3Sc00072_G_0.52_1 | 2 |
| GH20 | PbPT3Sc00011_S_14.157_1 PbPT3Sc00026_Am_5.65_1 PbPT3Sc00026_Am_5.70_1 | 3 |
| GH31 | PbPT3Sc00024_G_0.33_1 PbPT3Sc00040_A_1.338_1 | 2 |
| GH37 | PbPT3Sc00031_A_12.282_1 | 1 |
| GH38 | PbPT3Sc00016_Sm_4.295_1 PbPT3Sc00030_S_4.193_1 PbPT3Sc00072_Am_1.107_1 | 3 |
| GH45 | PbPT3Sc00026_Am_1.188_1 | 1 |
| GH47 | PbPT3Sc00011_A_1.275_1 PbPT3Sc00031_A_9.331_1 PbPT3Sc00039_Am_8.159_1 | 3 |
| GH49 | PbPT3Sc00030_Am_10.180_1 | 1 |
| GH63 | PbPT3Sc00092_S_4.265_1 | 1 |
| GH71 | PbPT3Sc00003_Am_0.143_1 | 1 |
| GH74 | PbPT3Sc00018_Am_2.114_1 | 1 |
| GH89 | PbPT3Sc00011_Am_2.127_1 | 1 |
| GH99 | PbPT3Sc00072_Am_0.117_1 | 1 |
| GH108 | PbPT3Sc00086_Am_6.45_1 | 1 |
| GH109 | PbPT3Sc00011_S_11.333_1 | 1 |
| GH113 | PbPT3Sc00057_Am_2.190_1 | 1 |
| GH114 | PbPT3Sc00036_Am_1.105_1  PbPT3Sc00036_Am_4.150_1  PbPT3Sc00036_Am_4.84_1  PbPT3Sc00036_Sm_4.191_1  PbPT3Sc00039_Am_10.129_1  PbPT3Sc00039_Am_11.196_1  PbPT3Sc00039_Sm_11.208_1  PbPT3Sc00039_Sm_11.249_1  PbPT3Sc00039_Sm_11.255_1  PbPT3Sc00039_Sm_11.257_1  PbPT3Sc00039_Sm_11.273_1  PbPT3Sc00058_Am_2.176_1  PbPT3Sc00070_A_0.264_1  PbPT3Sc00070_Am_0.111_1  PbPT3Sc00070_Am_0.115_1  PbPT3Sc00086_Sm_1.265_1  PbPT3Sc00097_Am_0.60_1 | 17 |
| GH125 | PbPT3Sc00039_A_1.296_1 PbPT3Sc00039_A_1.296_1 | 2 |
| GH131 | PbPT3Sc00024_Am_2.121_1 | 1 |
| **Glycosyltransferases** | | **108** |
| GT1 | PbPT3Sc00030_Am_4.80_1 PbPT3Sc00058_Am_2.119_1 | 2 |
| GT2 | PbPT3Sc00011_A_12.310_1 PbPT3Sc00011_Am_9.213_1 PbPT3Sc00011_Sm_3.201_1 PbPT3Sc00030_Am_8.185_1 PbPT3Sc00030_S_5.339_1 PbPT3Sc00031_Am_9.183_1 PbPT3Sc00047_S_6.317_1 PbPT3Sc00059_Am_6.82_1 PbPT3Sc00088_S_1.357_1 PbPT3Sc00092_G_1.107_1 PbPT3Sc00092_S_5.276_1 PbPT3Sc00103_A_0.270_1 | 12 |
| GT4 | PbPT3Sc00011_G_4.59_1 PbPT3Sc00011_S_6.367_1 PbPT3Sc00047_S_6.317_1 PbPT3Sc00048_Am_0.194_1 PbPT3Sc00069_S_0.237_1 | 5 |
| GT7 | PbPT3Sc00024_Am_1.180_1 | 1 |
| GT8 | PbPT3Sc00011_Sm_10.291_1 PbPT3Sc00033_Am_4.174_1 PbPT3Sc00059_Am_0.97_1 PbPT3Sc00072_Am_3.135_1 | 4 |
| GT10 | PbPT3Sc00026_Am_2.138_1 PbPT3Sc00031_A_3.208_1 PbPT3Sc00031_Am_8.174_1 PbPT3Sc00047_Am_0.124_1 | 4 |
| GT11 | PbPT3Sc00069_Am_2.136_1 | 1 |
| GT12 | PbPT3Sc00011_Sm_5.292_1 | 1 |
| GT13 | PbPT3Sc00048_Am_6.107_1 PbPT3Sc00074_A_0.271_1 | 2 |
| GT17 | PbPT3Sc00077_Am_0.139_1 PbPT3Sc00077_Am_1.91_1 | 2 |
| GT18 | PbPT3Sc00030_Am_1.107_1 | 1 |
| GT20 | PbPT3Sc00016_A_8.299_1 PbPT3Sc00018_Sm_4.147_1 | 2 |
| GT21 | PbPT3Sc00016_Am_9.156_1 PbPT3Sc00105_Am_1.113_1 | 2 |
| GT22 | PbPT3Sc00011_A_2.346_1 PbPT3Sc00030_Am_8.123_1 PbPT3Sc00060_Am_2.85_1 PbPT3Sc00066_Sm_1.273_1 | 4 |
| GT23 | PbPT3Sc00030_S_12.272_1 PbPT3Sc00041_Am_5.46_1 PbPT3Sc00046_Am_0.128_1 PbPT3Sc00059_Am_0.121_1 | 4 |
| GT24 | PbPT3Sc00059_S_1.301_1 | 1 |
| GT25 | PbPT3Sc00036_Sm_4.224_1 | 1 |
| GT26 | PbPT3Sc00011_Sm_9.289_1 | 1 |
| GT28 | PbPT3Sc00011_Am_6.156_1 | 1 |
| GT32 | PbPT3Sc00011_Sm_10.291_1 PbPT3Sc00023_Sm_7.177_1 PbPT3Sc00026_Sm_3.293_1 PbPT3Sc00030_Am_8.192_1 PbPT3Sc00031_Sm_18.219_1 PbPT3Sc00031_Sm_18.297_1 PbPT3Sc00048_Am_6.100_1 PbPT3Sc00053_Am_0.83_1 | 8 |
| GT33 | PbPT3Sc00018_A_2.162_1 | 1 |
| GT34 | PbPT3Sc00060_Am_1.158_1 | 1 |
| GT47 | PbPT3Sc00041_Am_4.113_1 PbPT3Sc00088_Sm_0.145_1 | 2 |
| GT48 | PbPT3Sc00047_S_0.207_1 | 1 |
| GT50 | PbPT3Sc00039_G_4.102_1 | 1 |
| GT54 | PbPT3Sc00018_A_3.298_1 | 1 |
| GT57 | PbPT3Sc00030_A_5.182_1 PbPT3Sc00059_S_6.236_1 | 2 |
| GT58 | PbPT3Sc00058_S_8.298_1 | 1 |
| GT59 | PbPT3Sc00018_S_4.231_1 | 1 |
| GT60 | PbPT3Sc00011_Am_10.194_1  PbPT3Sc00016_Am_12.155_1  PbPT3Sc00016_G_9.89_1  PbPT3Sc00023_Am_0.88_1  PbPT3Sc00023_Sm_1.246_1  PbPT3Sc00033_Am_4.131_1  PbPT3Sc00057_Am_1.115_1  PbPT3Sc00095_S_0.225_1 | 8 |
| GT61 | PbPT3Sc00039_Am_2.105_1 PbPT3Sc00069_Sm_0.192_1 PbPT3Sc00072_A_3.263_1 PbPT3Sc00072_Sm_3.181_1 PbPT3Sc00092_Am_1.182_1 PbPT3Sc00030_Sm_1.243_1 | 6 |
| GT62 | PbPT3Sc00040_Sm_3.214_1 | 1 |
| GT64 | PbPT3Sc00060_Sm_0.196_1 | 1 |
| GT66 | PbPT3Sc00055_Am_0.130_1 PbPT3Sc00059_Am_0.109_1 PbPT3Sc00108_Am_4.134_1 | 3 |
| GT68 | PbPT3Sc00108_Am_4.134_1 | 1 |
| GT69 | PbPT3Sc00040_A_5.302_1 PbPT3Sc00040_A_7.214_1 PbPT3Sc00055_Am_0.151_1 PbPT3Sc00057_S_2.317_1 | 4 |
| GT71 | PbPT3Sc00030_Am_1.151_1 PbPT3Sc00043_Am_0.82_1 PbPT3Sc00050_Am_0.122_1 | 3 |
| GT74 | PbPT3Sc00059_A_3.242_1 | 1 |
| GT75 | PbPT3Sc00048_Am_6.131_1 | 1 |
| GT76 | PbPT3Sc00031_S_0.157_1 | 1 |
| GT77 | PbPT3Sc00039_Am_7.94_1 PbPT3Sc00039_Am_7.98_1 PbPT3Sc00059_Am_0.125_1 PbPT3Sc00059_Am_0.129_1 PbPT3Sc00059_Sm_0.240_1 | 5 |
| GT83 | PbPT3Sc00042_Am_0.86_1 | 1 |
| GT90 | PbPT3Sc00069_Am_0.111_1 PbPT3Sc00086_S_1.309_1 | 2 |
| **Polysaccharide lyases** | | **3** |
| PL7 | PbPT3Sc00011_Am_7.175_1 PbPT3Sc00048_Am_1.127_1 PbPT3Sc00048_Am_1.131_1 | 3 |
| Total number | | 298 |
