# Supplementary material for: The compact genome of the plant pathogen Plasmodiophora brassicae is adapted to intracellular interactions with host Brassica spp
Source: BMC Genomics. 2016 Mar 31;17:272. doi: 10.1186/s12864-016-2597-2 (PMC4815078; doi:10.1186/s12864-016-2597-2)
Supplement: Additional file 15: Table S7. — Biosynthetic pathways for amino acids and their relative completeness in the P. brassicae genome. (DOCX 14 kb) [file 12864_2016_2597_MOESM15_ESM.docx]

**Additional file 15**

#### Table S7 Biosynthetic pathways for amino acids and their relative completeness in the P. brassicae genome.

| Absent | |
| --- | --- |
| His Ile Leu Trp Val | |
|  | |
| Partial | |
| Thr | Threonine synthase was not found by automated analysis but could be found manually (PbPT3Sc00036_A_3.327_1). However, homoserine kinase could not be found in the transcripts or genome. |
| Met | Missing cystathionine beta –lyase |
| Arg | Three enzymes for conversion of glutamate to ornithine are absent |
| Lys | The pathway via tetrahydrodipiconlinate is absent. Synthesis via the saccharopine pathway may be possible. |
|  | |
| Present | |
| Ala Asp Cys Gln Glu Gly Pro Ser Shikimic acid pathway | |
| Tyr | Prephenate dehydrogenase activity is found in the bifunctional chorismate synthase that catalyses the previous step |
| Phe | The final step is an aromatic amino acid transferase which has not been identified but may be catalyzed by related enzymes |
|  |  |
| Unknown |  |
| Asn | The enzyme catalyzing asparagine synthesis has not been identified in many organisms |
