# Supplementary material for: The compact genome of the plant pathogen Plasmodiophora brassicae is adapted to intracellular interactions with host Brassica spp
Source: BMC Genomics. 2016 Mar 31;17:272. doi: 10.1186/s12864-016-2597-2 (PMC4815078; doi:10.1186/s12864-016-2597-2)
Supplement: Additional file 16: Table S8. — Putative amino acid transporters. (DOCX 14 kb) [file 12864_2016_2597_MOESM16_ESM.docx]

**Additional file 16**

#### Table S8 Putative amino acid transporters.

| **Gene ID** | **E-value** | **Best match to TCDB** |
| --- | --- | --- |
| PbPT3Sc00024_A_0.263_1 | 2E-44 | C4LSN3 2.A.18.10.2 Amino acid transporter, putative  OS=Entamoeba histolytica GN=EHI_151940 PE=4 SV=1 |
| PbPT3Sc00030_S_2.309_1 | 2E-20 | Q5C8V6 2.A.3.12.1 Polyamine transporter (Amino acid  permease, putative) - Leishmania major. |
| PbPT3Sc00031_A_16.253_1 | 1E-32 | Q9JM15 2.A.18.6.1 NEURONAL GLUTAMINE TRANSPORTER -  Rattus norvegicus (Rat). |
| PbPT3Sc00058_A_8.292_1 | 4E-17 | Q19834 2.A.3.8.9 Hypothetical protein aat-1 - Caenorhabditis  elegans. |
| PbPT3Sc00066_Am_1.163_1 | 8E-43 | Q9UT18 2.A.3.4.6 Uncharacterized amino-acid permease  C9.10 - Schizosaccharomyces pombe (Fission yeast). |
| PbPT3Sc00059_Am_6.118_1 | 1E-32 | P36062 2.A.18.7.1 Hypothetical 75.5 kDa protein in  SDH1-CIM5/YTA3 intergenic region - Saccharomyces cerevisiae  (Baker's yeast) |
| PbPT3Sc00024_Am_1.105_1 | 3E-42 | Q9WTR6 2.A.3.8.5 Cystine/glutamate transporter (Amino acid transport system xc-) (xCT) - Mus musculus (Mouse). |
| PbPT3Sc00053_Am_1.102_1 | 9E-24 | Q9DCP2 2.A.18.6.2 2.A.18.6.2 Sodium-coupled neutral  amino acid transporter 3 |
| PbPT3Sc00058_S_8.315_1 | 1E-19 | Q19834 2.A.3.8.9 Hypothetical protein aat-1 - Caenorhabditis  elegans. |
| PbPT3Sc00097_G_2.73_1 | 2E-61 | P82252 2.A.3.8.15 B(0,+)-type amino acid transporter  1 - Rattus norvegicus (Rat). |
